# Frontoparietal network activation is associated with motor recovery in ischemic stroke patients

**DOI:** 10.1038/s42003-022-03950-4

**Published:** 2022-09-21

**Authors:** Emily Olafson, Georgia Russello, Keith W. Jamison, Hesheng Liu, Danhong Wang, Joel E. Bruss, Aaron D. Boes, Amy Kuceyeski

**Affiliations:** 1 Department of Radiology, Weill Cornell Medicine, New York City, NY 10021 USA; 2 Pelham Memorial High School, 575 Colonial Ave, Village of Pelham, NY 10803 USA; 3 Department of Radiology, Harvard Medical School, Boston, MA 02115 USA; 4 Department of Neurology, University of Iowa, Iowa City, IA 52242 USA

**Keywords:** Dynamical systems, Stroke

## Abstract

Strokes cause lesions that damage brain tissue, disrupt normal brain activity patterns and can lead to impairments in motor function. Although modulation of cortical activity is central to stimulation-based rehabilitative therapies, aberrant and adaptive patterns of brain activity after stroke have not yet been fully characterized. Here, we apply a brain dynamics analysis approach to study longitudinal brain activity patterns in individuals with ischemic pontine stroke. We first found 4 commonly occurring brain states largely characterized by high amplitude activations in the visual, frontoparietal, default mode, and motor networks. Stroke subjects spent less time in the frontoparietal state compared to controls. For individuals with dominant-hand CST damage, more time spent in the frontoparietal state from 1 week to 3-6 months post-stroke was associated with better motor recovery over the same time period, an association which was independent of baseline impairment. Furthermore, the amount of time spent in brain states was linked empirically to functional connectivity. This work suggests that when the dominant-hand CST is compromised in stroke, resting state configurations may include increased activation of the frontoparietal network, which may facilitate compensatory neural pathways that support recovery of motor function when traditional motor circuits of the dominant-hemisphere are compromised.

## Abstract (web summary)

Advanced neuroimaging methods in stroke patients demonstrate that the more stroke survivors with dominant hemisphere corticospinal tract damage recruit their brain frontoparietal network, the better their motor recovery.

## Introduction

The ability to perform motor functions after stroke depends on the coordinated reconfiguration of distinct global brain activity patterns^1^. Novel data-driven techniques to characterize whole-brain activity in functional magnetic resonance imaging (fMRI) scans at single-frame resolutions have illuminated the dynamic nature of brain activity in several brain disorders^2–5^, but methods assigning each time point to a discrete state have not yet been applied to examine altered brain dynamics in stroke patients. These methods provide information about brain function complementary to and beyond traditional static measures of functional connectivity (FC)^6^, and thus may provide new insights regarding the process of recovery following stroke.

Prior work characterizing spatiotemporal brain dynamics after stroke has focused on identifying altered FC states, which reflect time-varying patterns of FC. Dynamic FC analyses identify recurrent connectivity patterns using a sliding-window approach, in which FC is repeatedly calculated over consecutive windowed segments of the fMRI scan. This approach yields FC networks that fluctuate over time, with a temporal resolution proportional to the size of the window; ~30–60 seconds^7^. In stroke populations, dynamic FC studies have demonstrated stroke-related differences in temporal configurations of motor networks^8^ and participation in connectivity states that varies with severity^9^.

In contrast, analysis at a single relaxation time (TR) resolution of activation states identified in a data-driven fashion using k-means clustering of the time series data^6^ provides a closer look at the moment-to-moment changes in recurrent brain activity, with the time spent in each state lasting, on average, 5–10 seconds^6^. A benefit to analyzing brain activation states over or alongside connectivity states is that activation patterns can enable a more refined interpretation of connectivity differences between groups. FC is traditionally defined as the correlation between two brain region’s activity over time. FC may be driven by two distinct features of brain activity: by the individualized spatial patterns of large-amplitude activations^10^, and by the amount of time spent in recurring patterns of activity^11,12^. In this paper, we aim to identify group-level patterns of brain activity after stroke that relate to recovery, and assume that recurring activity patterns are shared across individuals but are expressed in different proportions. Understanding the temporal patterns of activity underlying recovery-relevant FC changes after stroke can aid in the development of more accurate targets in stimulation therapies.

Recent work has highlighted the importance of frontoparietal areas in supporting motor abilities in the chronic phase of stroke^13,14^ specifically in patients with poor corticospinal tract (CST) integrity^15^. When these descending motor pathways are significantly damaged, descending white matter tracts from higher-order motor areas, like regions of the frontoparietal network (FPN), may support motor output. Because of the differential use of the dominant and non-dominant arm throughout life, we were interested in determining whether handedness relative to the lesioned hemisphere would modify the recruitment of a frontoparietal state to promote motor recovery. Understanding this type of subject- and lesion-specific variability in the post-stroke recovery process is precisely the type of information needed to develop personalized rehabilitation strategies to maximally promote recovery.

Here, we propose to first identify and characterize recurring brain activity patterns, or states, in healthy controls and individuals with ischemic pontine stroke^6^. We hypothesized that individuals with ischemic stroke would display altered dynamic brain state metrics, e.g. fractional occupancy (FO), dwell time (DT), and appearance rates (ARs), compared to control subjects, and, further, that these dynamic state metrics would be associated with measures of motor recovery. In an exploratory analysis, we examined whether time spent in a brain state characterized by frontoparietal activation would be differentially recruited for later-stage motor recovery depending on the side of the lesion relative to the subject’s handedness. Finally, to bridge dynamic brain state analyses and more classic FC approaches, we assessed the relationship between the amount of time spent in different brain states and the FC between several resting-state networks. This last analysis is particularly important in terms of linking our current findings to previous studies of how rehabilitation techniques, including non-invasive brain stimulation, modulate the functional connectome and possibly motor recovery.

## Results

### Participant characteristics

Differences in the age- and sex- composition of the stroke and control group were compared with a two-tailed unpaired *t* test and Fisher’s exact test, respectively (Table 1).

**Table 1:** Demographic and clinical characteristics of sample.

Group	Total No.	Age [median]	Sex F	Sex M	FM1	FM1	FM3	FM4	FM5
Stroke	23	34–74 [57]	8	15	54.3 (33.1)	70.3 (28.8)	80.7 (22.7)	87.9 (16.2)	91.8 (11.4)
Control	24	33–65 [52.5]	10	14					
Differences*		−2.105 (0.041)	0.746 (0.766)						

*Differences between groups. For age: unpaired two-tail *t* test; *t* statistic (*p* value), for sex: Fisher’s exact test: odds ratio (*p* value). Motor scores: *FM1* Fugl-Meyer score at 1-week post stroke, *FM2* Fugl-Meyer score at 2 weeks post stroke, *FM3* Fugl-Meyer score at 1-month post stroke, *FM4* Fugl-Meyer score at 3 months post stroke, *FM5* Fugl-Meyer score at 6 months post stroke. Displayed as: mean (standard deviation).

### Clustering reveals an optimum of four brain states

**Fig. 1:**
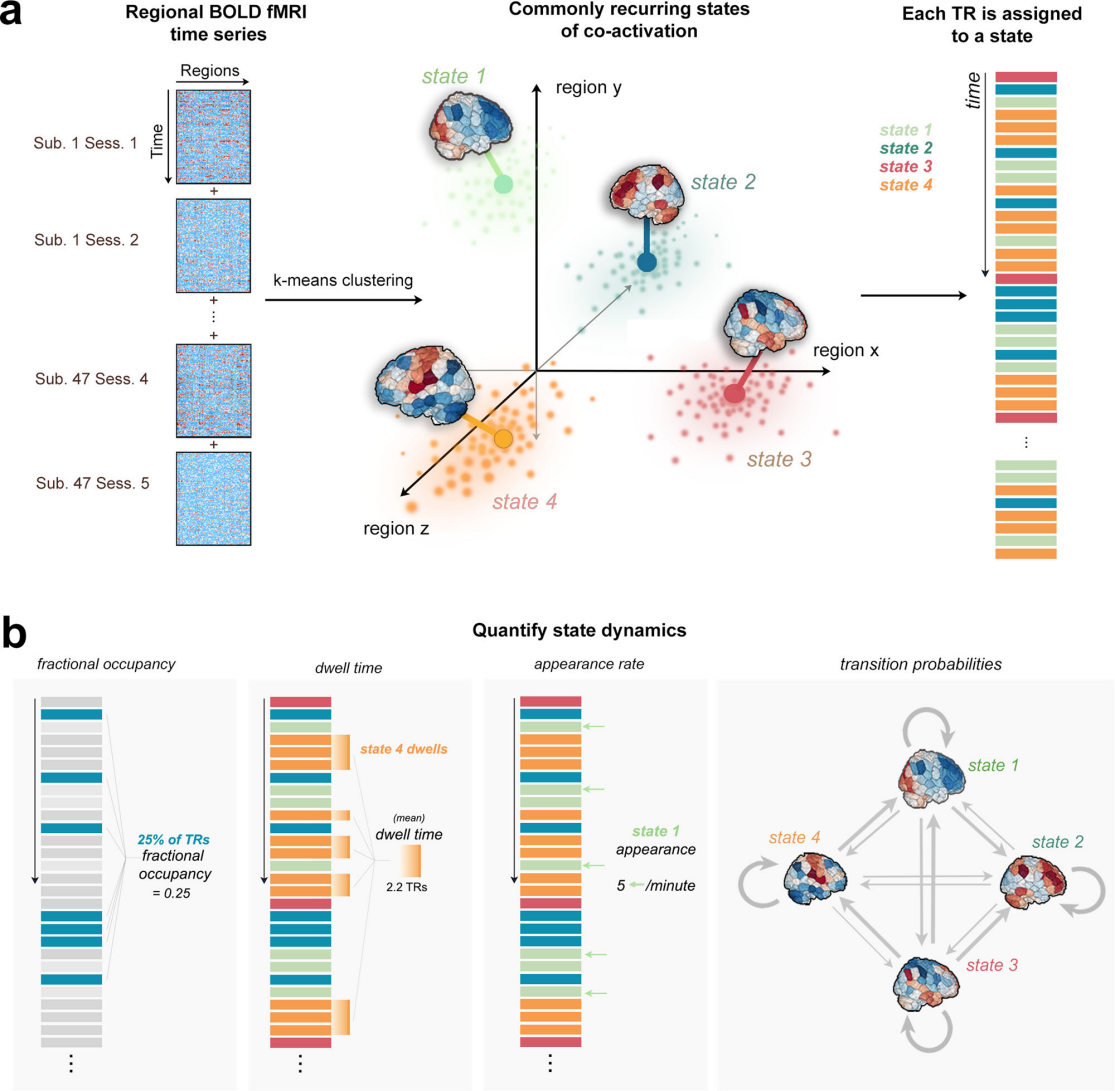
Clustering of time series data and quantification of dynamic state metrics. **a** Time series data from all subjects were concatenated together along the time dimension. K-means clustering produced 4 distinct brain activation states defined by different locations in regional activation space (image adapted from ref. ^6^). Each TR is assigned to one of four brain states based on k-means partitions. **b** Fractional occupancy, dwell time, appearance rate, and transition probabilities are calculated separately for each subject and for each state.

We first clustered the data into discrete clusters using the *k*-means algorithm (Fig. 1). To identify the optimal number of clusters (*k*), we observed the change in variance explained with increasing *k* (Supplementary Fig. 5a); the difference in variance explained between *k* = 4 and *k* = 5 was <2%. The elbow of the curves were between 4 and 6 clusters across the cluster quality metrics (Supplementary Fig. 5c). We chose *k* = 4 for parsimony, and replicate *k* = 5 in the supplemental information section (Supplementary Fig. 11). Silhouette values for *k* = 4 revealed good cluster assignment with very few TRs assigned erroneously (i.e., having negative Silhouette values) (Supplementary Fig. 5b). In general, we found that the mutual information shared between partitions was quite high (0.88–1), suggesting consistent clustering across independent initializations of k-means (Supplementary Fig. 5d). The four brain states found with the 268-region Shen atlas are replicated with 86 and 400 region atlases (Supplementary Fig. 7). Finally, the states identified by clustering stroke and control groups separately are similar to the states identified by clustering all individuals together (Supplementary Fig. 6). Therefore, we report the full clustering results in the main text and include the various replications in the Supplemental Information.

**Fig. 2:**
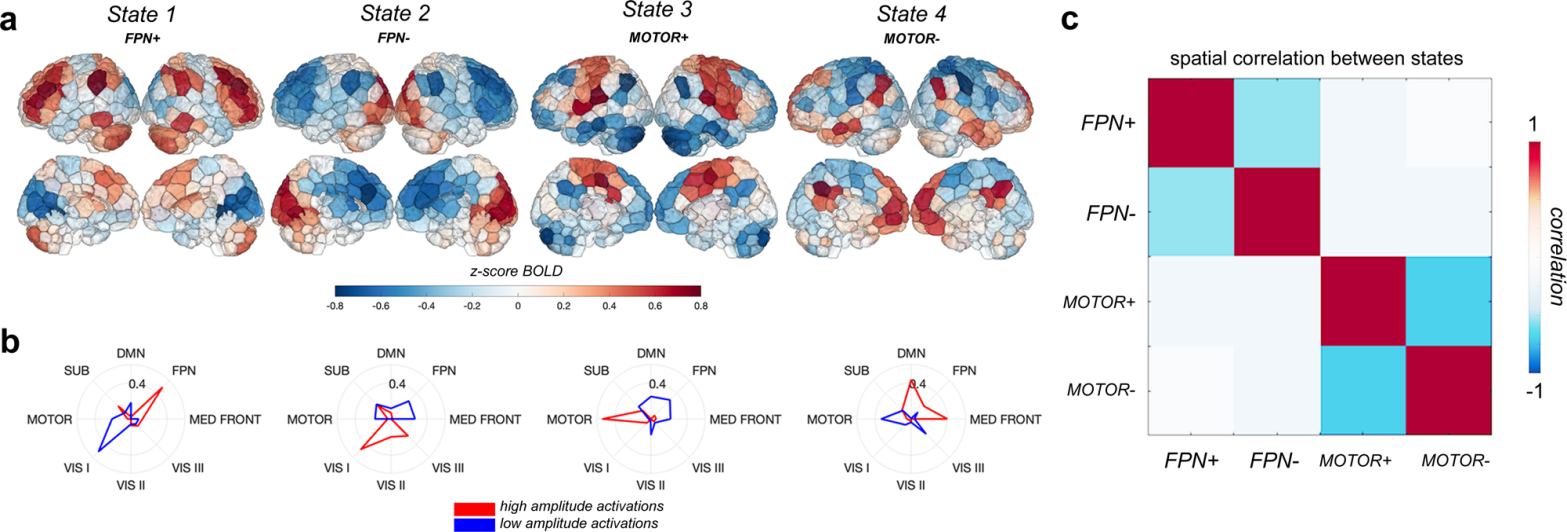
Four brain activity states. **a** Centroids of each state calculated as the mean of the normalized regional activation over all TRs assigned to that state. **b** Radial plots displaying cosine similarity between each cluster and each canonical network; red indicates cosine similarity of the high amplitude activations, blue indicates cosine similarity of the low amplitude activations. Labels for each state were derived from analyzing the magnitude and type of overlap of each centroid with the 8 canonical networks. **c** Region-level correlation of each pair of brain state centroids. DMN default mode network, FPN frontoparietal network, MED FRONT medial frontal network, VIS III visual association network, VIS II visual network 2, VIS I visual network 1, MOTOR motor network, SUB subcortical/cerebellum network.

The four brain states consist of distinct activation levels across the various canonical resting-state networks (Fig. 2a, b). State 1 was characterized by high amplitude activation of regions in the frontoparietal network and low amplitude activations in the visual I network (*FPN*^+^), state 2 by low amplitude activation in the frontoparietal network and high amplitude in the visual I network (*FPN*^−^) (equal magnitude activations), state 3 by high amplitude activation of the somatomotor network and low amplitude activation in the default mode network (*MOTOR*^+^), while state 4 was characterized by low amplitude activation of the motor network as well as high amplitude activation of the default mode network (*MOTOR*^−^). The correlations between the 268-region centroids of the *FPN*^+^ and *FPN*^−^ states and the *MOTOR*^+^ and *MOTOR*^−^ states were strong and negative, suggesting that brain states are likely hierarchically organized into two meta-states that are each composed of two sub-states containing opposing activation patterns (Fig. 2c), a finding which has been previously reported in other work using this technique^5^.

### Stroke-control differences in brain state dynamics

**Fig. 3:**
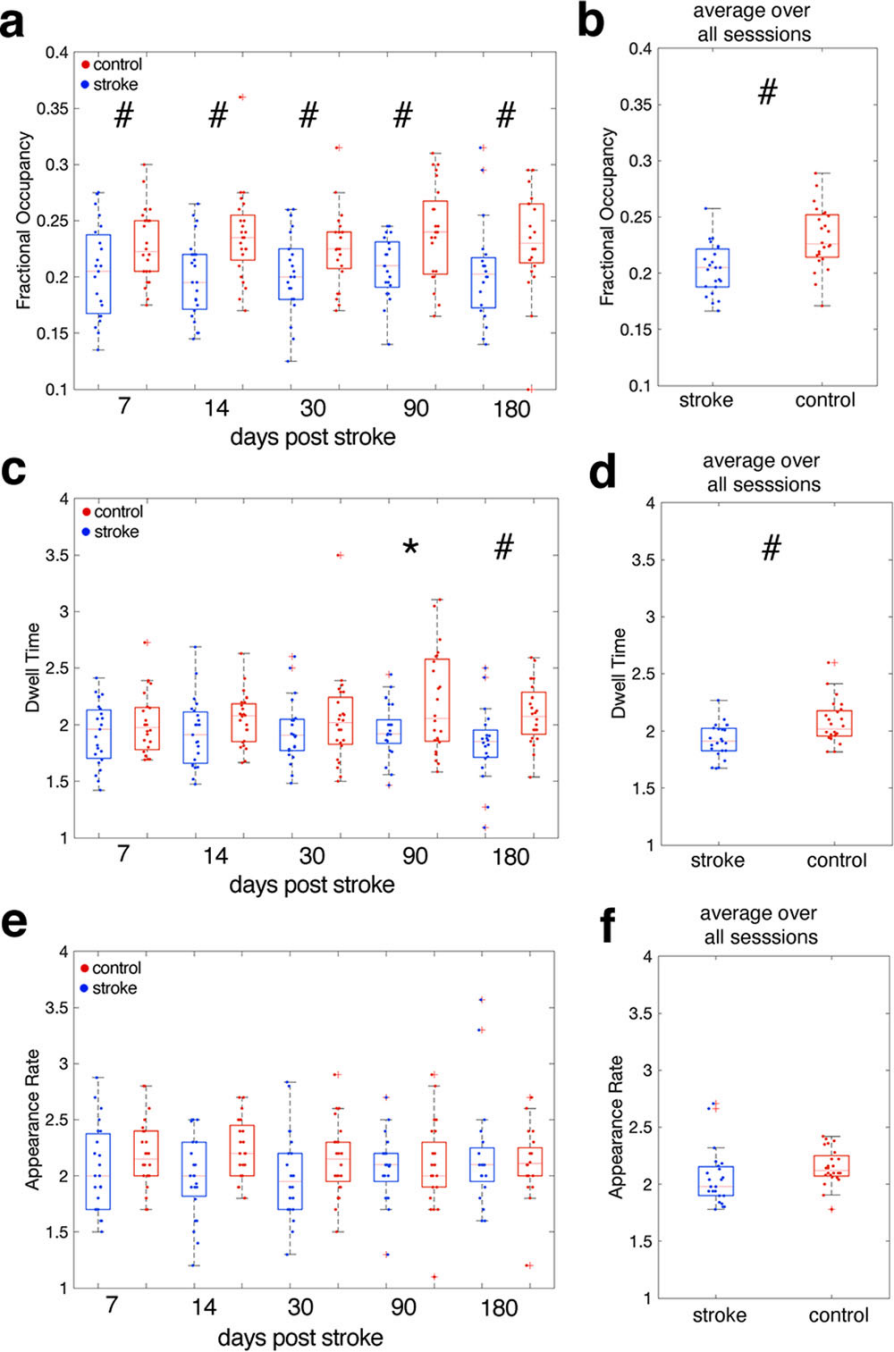
Group differences in brain state dynamics for *FPN*^+^. **a**, **c**, **e** Stroke-control differences in session-specific fractional occupancy (FO), dwell time (DT), and appearance rate (AR) in *FPN*^+^ over the post-stroke recovery period. **c**, **d**, **f** Stroke-control differences in average FO, DT, and AR (averaged over each subject's 2–5 longitudinal sessions). Hashtags (*#*) represent *p* values < 0.05 after multiple comparisons correction within each parameter (i.e., FO, DT, AR), single asterisks (*) represent significant uncorrected *p* values < 0.05. Dwell time shown in units of TRs. *n* = 24 biologically independent control subjects at each time point; *n* = 23, 23, 22, 21, 20 biologically independent stroke subjects for time point 1, 2, 3, 4, and 5, respectively. On each box, the central mark indicates the median, and the bottom and top edges of the box indicate the 25th and 75th percentiles, respectively.

FO, DT, and AR of each state were calculated for each subject (Fig. 1a, b). Because the control subjects were younger on average than the stroke subjects, we assessed the correlation between age and brain state dynamic parameters in both groups (Supplementary Fig. 9) and determined that there was no relationship between age and any parameter for any state for stroke or control subjects. The average FO, DT, and AR across all available time points (1 week, 2 weeks, 1 month, 3 months, and 6 months) for each of the four states were compared between stroke and control subjects via unpaired two-tail t-tests (Fig. 3, see Supplementary Fig. 8 for full session-specific results for each state, and see Supplementary Fig. 10 for the general pattern of brain dynamics observed in stroke and control subjects). Significantly lower FO of *FPN*^+^ in stroke subjects was observed at every session from 1 week to 6 months post-stroke (session 1 t-statistic: −2.18, 95% CI = [−0.0587, −0.0019], session 2 t-statistic: −3.36, 95% CI = [−0.0458, −0.014], session 3 t-statistic: −2.24, 95% CI = [−0.0435, −0.0023], session 4 t-statistic: −2.14, 95% CI = [−0.0516, −0.0016], session 5 t-statistic: −2.0725, 95% CI = [−0.0553, −0.0007], p(FDR) for all five tests < 0.05) (Fig. 3a). Differences in DT of *FPN*^+^ were observed only in the chronic stage of stroke (6 months post-stroke) (p(FDR) = 0.0315, corrected over five tests) (Fig. 3c). Stroke subjects had significantly lower time-averaged FO in *FPN*^+^ compared to control subjects ((t-statistic: −3.7334, 95% CI = [−0.0425, −0.0127], p(FDR) = 0.0021), which was possibly driven more by the significantly lower DTs in *FPN*^+^ observed in stroke subjects (p(FDR) = 0.0105) (Fig. 3b, d). The frontoparietal network used in this atlas contains nodes in the dorsolateral prefrontal cortex, posterior parietal cortex, as well as nodes in the posterior inferior temporal lobe and inferior cerebellum. No differences in AR of *FPN*^+^ were observed over the sessions (Fig. 3e, f).

**Fig. 4:**
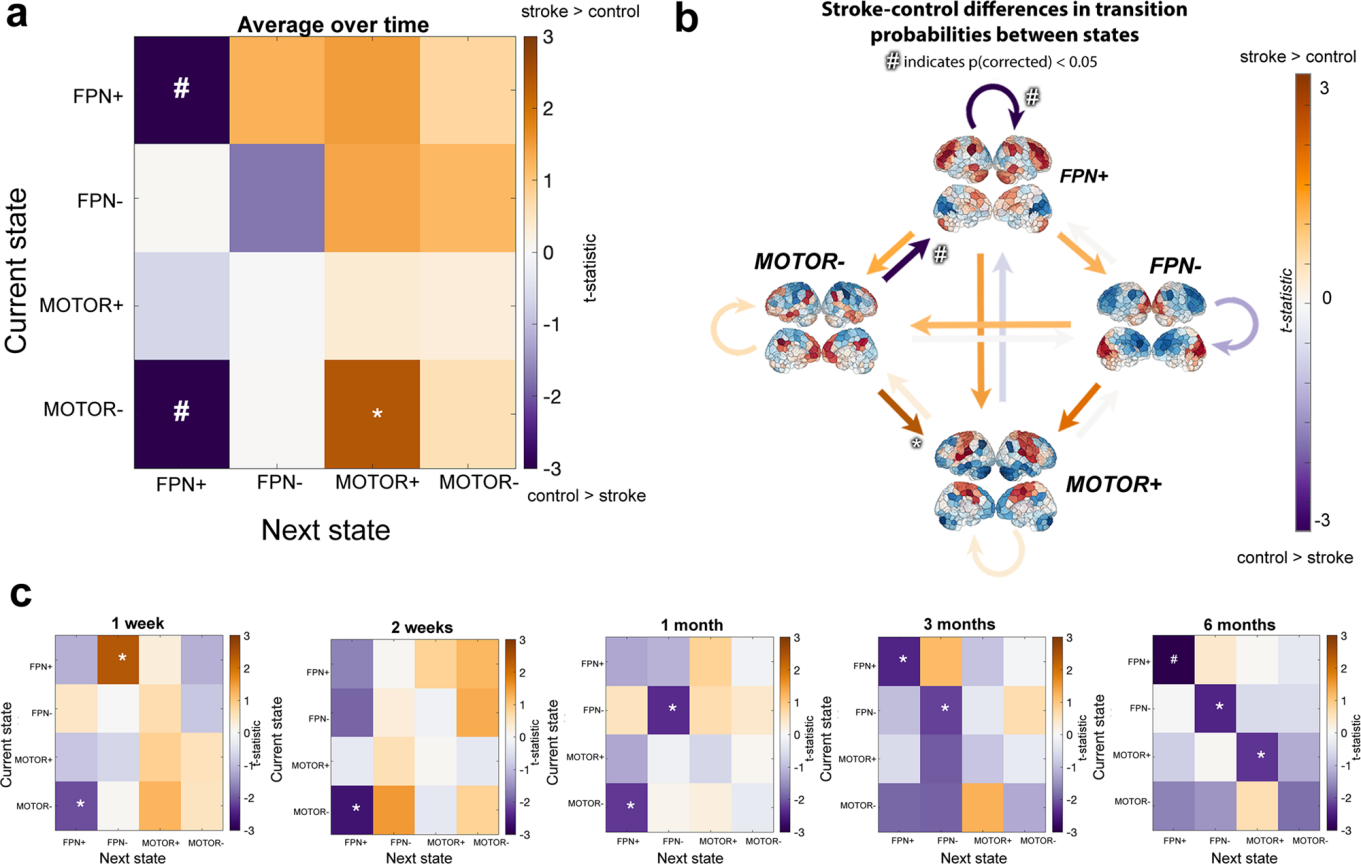
Differences in transition probabilities between stroke and control groups. Transition probabilities include persistence probabilities, i.e. the probability that a state does not transition out of itself. **a** Differences in average transition and persistence probabilities (over time) between groups. **b** Same data as **a**, visualized with arrows colored by t-statistic. **c** Differences in transition probabilities at each follow-up session. T-statistics (stroke-control) are displayed on the color map; hashtags (*#*) represent *p* values < 0.05 after multiple comparisons correction, single asterisks (*) represent significant uncorrected *p* values < 0.05. *n* = 24 biologically independent control subjects at each time point; *n* = 23, 23, 22, 21, 20 biologically independent stroke subjects for time point 1, 2, 3, 4, and 5, respectively.

Stroke subjects had significantly reduced transition probabilities from *FPN*^+^ and *MOTOR*^−^ into *FPN*^+^ (p(FDR) = 0.023 and 0.020, respectively) (Fig. 4a, b). The lower persistence probability in stroke for *FPN*^+^ seems to be driven by chronic-stage differences; in the session-specific analysis, individuals with stroke have a significantly lower persistence probability for *FPN*^+^ at the 6-month time point (p(FDR) = 0.030) and there is a trend for lower persistence probability at 3 months (Fig. 4c).

### Frontoparietal activation relates to motor recovery in individuals with dominant-hand CST damage

**Fig. 5:**
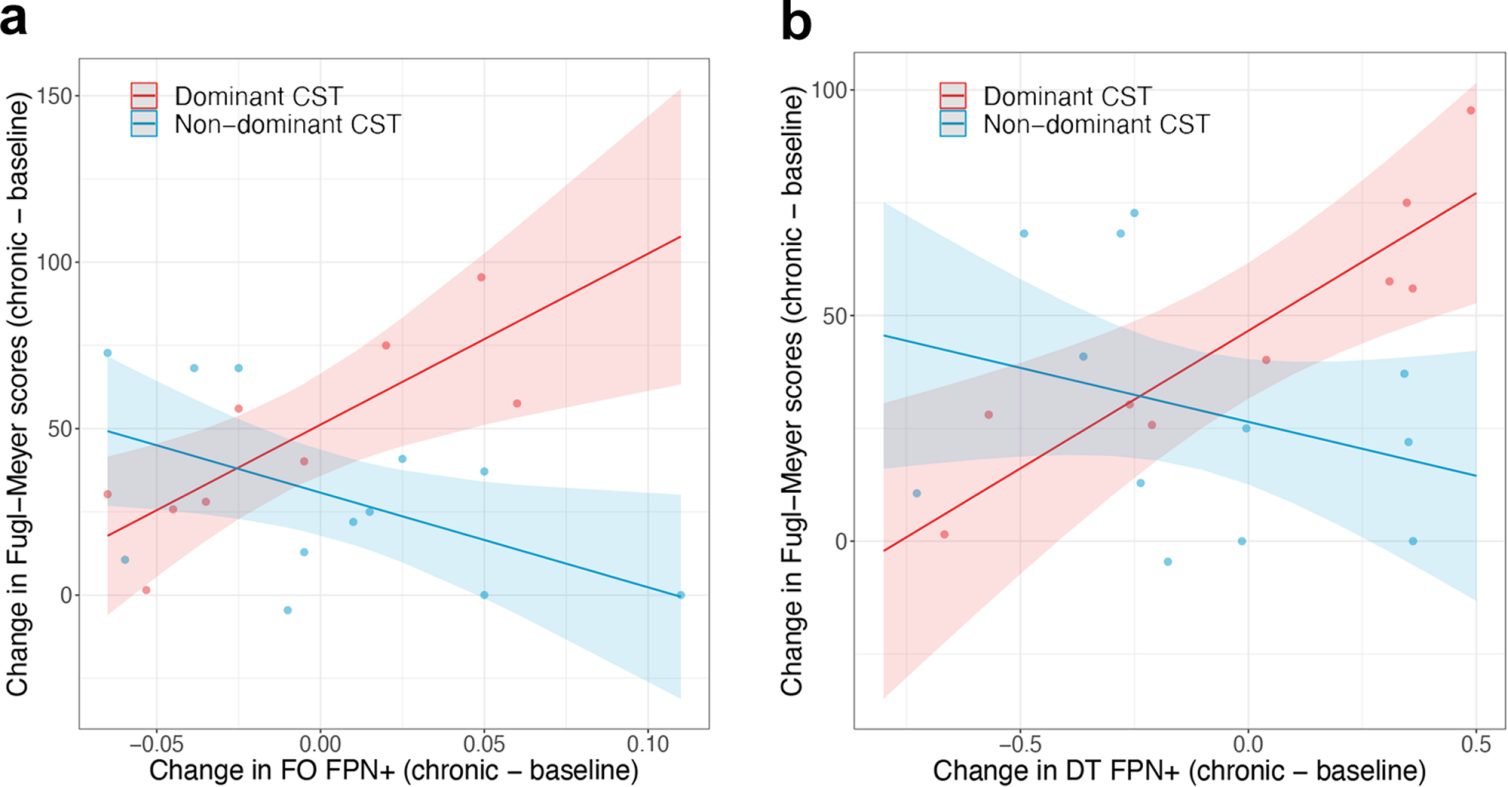
Results from linear models with N = 21 for each model. Colors indicate the marginal effects plots of change in state metrics within the regression model. Marginal effects plots of the change in Fugl–Meyer scores versus the change in fractional occupancy (FO) (**a**) and dwell time (DT) (**b**) of *FPN*^+^ from baseline to chronic time points. Shaded bars indicate 95% confidence intervals.

We observed a significant interaction effect between changes in *FPN*^+^ parameters and dominant-hand CST damage on changes in Fugl−Meyer scores ($CS{T}_{D}* \Delta F{O}_{chronic}^{FP{N}^{+}}$: p(FDR) = 0.0091, $CS{T}_{D}* \Delta F{O}_{chronic}^{FP{N}^{+}}$: p(FDR) = 0.0090), as well as a significant association between changes in *FPN*^+^ parameters and changes in Fugl-Meyer scores for subjects with dominant-hand CST damage (i.e., marginal effects) ($\Delta D{T}_{chronic}^{FP{N}^{+}}$: p(FDR) = 0.0091, $\Delta F{O}_{chronic}^{FP{N}^{+}}$: p(FDR) = 0.0098), such that increases in (or smaller decreases in) DTs in these subjects were associated with greater motor improvements (Fig. 5). We confirmed the accuracy of our models by verifying that the residuals were normally distributed (Supplementary Fig. 13). Some individuals with dominant-hand CST damage show decreases in *FPN*^+^ recruitment over time, but smaller decreases in $D{T}^{FP{N}^{+}}$ and $F{O}^{FP{N}^{+}}$ are associated with better recovery. Including age and sex in the models does not alter the results ($CS{T}_{D}* \Delta F{O}_{chronic}^{FP{N}^{+}}$: p(FDR) = 0.0166, $CS{T}_{D}* \Delta D{T}_{chronic}^{FP{N}^{+}}$: p(FDR) = 0.0301, marginal $\Delta F{O}_{chronic}^{FP{N}^{+}}$: p(FDR) = 0.016, $\Delta D{T}_{chronic}^{FP{N}^{+}}$: p(FDR) = 0.016). The marginal effect for the DT analysis was replicated in *k* = 5 (Supplementary Fig. 11) but not the interaction effect, nor either effect for FO.

Finally, we observe that there was a strong and significant correlation between baseline FM and $\Delta D{T}_{chronic}^{FP{N}^{+}}$ and $\Delta F{O}_{chronic}^{FP{N}^{+}}$ for subjects with dominant-hand CST damage (*R* = −0.83, *p* = 0.0054, *R* = −0.71, *p* = 0.033, respectively), see (Supplementary Fig. 14a, b), such that subjects with more baseline impairment had the greatest increases in $\Delta D{T}^{FP{N}^{+}}$ and $\Delta F{O}^{FP{N}^{+}}$ from baseline to chronic time points. Therefore, we proceeded with creating the null model as described in the Methods section to verify that the observed relationships between Δ*FM* and $\Delta DT/F{O}_{chronic}^{FP{N}^{+}}$ were not a byproduct of the correlation with baseline *FM*. Indeed, the observed correlation between Δ*FM* and $\Delta D{T}_{chronic}^{FP{N}^{+}}$ is significantly greater than the null distribution of correlation between simulated proportional recovery and $\Delta D{T}_{chronic}^{FP{N}^{+}}$ (*p* = 0.0240) (Supplementary Fig. 14c). However, the observed correlation between Δ*FM* and $\Delta F{O}_{chronic}^{FP{N}^{+}}$ is not significantly greater than what is observed in the null distribution (*p* = 0.50) (Supplementary Fig. 14d). This suggests that the observed relationship between Δ*FM* and $\Delta D{T}_{chronic}^{FP{N}^{+}}$ in subjects with dominant hemisphere CST damage is not merely due to baseline correlations and proportional recovery, whereas that the relationship between Δ*FM* and $\Delta F{O}_{chronic}^{FP{N}^{+}}$ may be driven by the degree of impairment at baseline.

### FO differences are related to FC differences

**Fig. 6:**
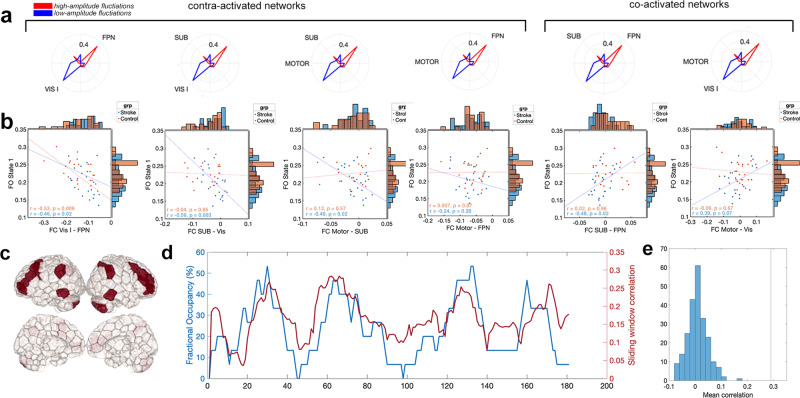
Assessing the relationship between fractional occupancy of *FPN*^+^ and functional connectivity within the frontoparietal network. **a** Pairs of highly activated networks in *FPN*^+^. **b** Functional connectivity (FC) between co-activated and contra-activated networks in *FPN*^+^ is related to fractional occupancy (FO) of *FPN*^+^ ($F{O}^{FP{N}^{+}}$) in both stroke and control subjects. Specifically, fewer TRs in a state with strongly contra-activated/co-activated networks results in smaller magnitude FC between those networks. **c** Areas with high activation in the *FPN*^+^ centroid. **d** Dynamic, sliding-window fluctuations in $F{O}^{FP{N}^{+}}$ correlate with dynamic FC between regions highly active in the FPN (areas in **c**). **e** Subject-average correlation between dynamic, sliding window $F{O}^{FP{N}^{+}}$ and FC of regions highly active in *FPN*^+^ (black line) and a null distribution of 100 subject-average correlations between dynamic, sliding-window $F{O}^{FP{N}^{+}}$ and FC in a set of randomly selected regions (blue histogram).

Four network pairs had a high magnitude contra-activation (large-amplitude activity in opposite directions) in the *FPN*^+^ state: visual I (VIS I) and frontoparietal (FPN), subcortical/cerebellum (SUB) and visual I (VIS I), motor (MOTOR) and subcortical/cerebellum (SUB), and motor (MOTOR) and frontoparietal (FPN) (Fig. 6a). Two pairs of networks had high magnitude co-activation (large-amplitude activity in the same direction) in the *FPN*^+^ state: SUB and FPN, and MOTOR and VIS I (Fig. 6a). For each individual, we correlated $F{O}^{FP{N}^{+}}$ with the FC between each pair of co-activated and contra-activated networks, expecting two trends. First, for the highly contra-activated network pairs, we expected a negative correlation between $F{O}^{FP{N}^{+}}$ and FC, as more time spent in *FPN*^+^ would make the correlation between those networks more negative. Second, for the highly co-activated network pairs, we expected a positive correlation between $F{O}^{FP{N}^{+}}$ and FC as more time spent in this state would make the correlation between those networks more positive. We did indeed observe the expected relationships for all pairs of networks (Fig. 6b), where the strongest relationships between FC and *FO* were observed for those network pairs that had the greatest recruitment in *FPN*^+^, as measured by cosine similarity with the *FPN*^+^ centroid (VIS I and FPN). Further, we see that the correlation between $F{O}^{FP{N}^{+}}$ and FC is preserved across and within the groups and is not driven by across-group differences (Simpson’s paradox). Finally, dynamic fluctuations in $F{O}^{FP{N}^{+}}$ track changes in dynamic FC within frontoparietal areas (Fig. 6c–e), where the average correlation across subjects between sliding-window FC and $F{O}^{FP{N}^{+}}$ is 0.29 (*p* = 0, assessed by permutation).

## Discussion

Large-scale brain activity patterns, or states, can be thought of as group-level temporal building blocks of canonical FC, reflecting sequences of activity that lie ‘under the hood’ of FC. Mapping the dynamics of these states offers a fine-grained view into shifts in the temporal sequences of neural activity after stroke that has not yet been explored. In the present study, we provide evidence that spatiotemporal brain dynamics, particularly in a state characterized by high-amplitude frontoparietal activity, are altered after pontine stroke for at least up to 6 months post-infarction. We further demonstrate that increased DT in this frontoparietal state is meaningfully related to better improvements in motor function for individuals with dominant-hand CST damage. Finally, we show a direct relationship between time spent in brain states and FC both in controls as well as individuals with stroke.

We observed that stroke subjects spent less time in a brain state characterized by high amplitude frontoparietal network activation, particularly in the sub-acute to chronic stages of stroke (3 and 6 months post-stroke). The frontoparietal network, containing nodes in the frontal cortex like the dorsal premotor cortex and in the parietal cortex like the posterior parietal cortex and intraparietal sulcus, is thought to act as a top–down influence on primary motor networks to control motor output^16^. Communication between nodes in the frontoparietal network is known to be important for computations involved in goal-directed movement, such as motor imagery^17^, prospective action judgements^18^, and generating appropriate hand positions to interact with objects^19^. Evidence of reduced FC within the frontoparietal network has been observed in subcortical stroke subjects^20^, as has reduced effective connectivity of the frontoparietal network on the motor network^21^. Our study extends this current knowledge by demonstrating that the influence of the frontoparietal network in stroke subjects may be weakened, with reduced time spent in states with high amplitude frontoparietal activity.

We did not observe changes in the FO, DT, or AR of the *MOTOR*^+^ or *MOTOR*^−^ states in stroke subjects, as one may anticipate after damage to the CST. Instead, we observed changes in the transition probabilities between *MOTOR*^−^ and *FPN*^+^, and *MOTOR*^−^ and *MOTOR*^+^. These may be more subtle impacts of CST disruption that change the sequence of states visited at rest. A lack of differences in the *MOTOR* states could also arise due to the fact that the method cannot detect differences in the magnitude of activations. A critical step in the method involves normalizing each region’s brain activity before clustering. This step would theoretically remove stroke-related differences in the magnitude of activation of motor areas that one may expect after CST damage. As the focus of our paper was to explore stroke-related changes in the temporal patterns of brain activity, we did not explore this possibility, but future work should examine changes in both the magnitude and temporal properties of these states after stroke.

Recent work has shown that individuals with CST damage (measured using motor-evoked potentials) have greater resting-state FC in the frontoparietal network compared to individuals without CST damage^15^. We observed that individuals with dominant-hand CST damage had a positive relationship between changes in motor recovery and changes in DTs in the *FPN*^+^ state from 1 week to the chronic time period post stroke. Increased time spent in the frontoparietal network in subjects with dominant hemisphere CST damage may be a form of compensation related to structural reserve capacity, a concept believed to reflect increased neural substrate that is neuroprotective against stroke and can modify outcomes^22^. This result, from an exploratory analysis, suggests that frontoparietal activation may be an adaptive strategy to support motor recovery which may be more relevant for subjects with dominant hemisphere CST damage. This hypothesis requires formal examination in future research.

Finally, we showed that across controls and individuals with stroke, FO of the *FPN*^+^ state and FC between networks that dominate that state were related. For example, FC between pairs of networks that were co-activated in the *FPN*^+^ state were positively correlated with FO and FC between pairs of networks that were contra-activated in the *FPN*^+^ state were negatively correlated with FO. Less negative FC between the visual and FPN network was related to less time spent in *FPN*^+^, a state in which the two networks had large magnitude activations in opposite directions. Our findings suggest that time spent in certain brain states may underlie between-group FC differences; a finding which we also replicated with dynamic, sliding-window analysis. Knowing how stroke and recovery from stroke are related to time spent in these states may be helpful in designing non-invasive stimulation strategies that are based on direct co-activation/conta-activation of certain regions or networks, not on indirect modulation of FC between pairs of regions/networks.

Consideration of state metrics like DTs, FO, AR, and transition probability has the potential to be of great clinical relevance. Stimulation therapies like transcranial magnetic stimulation (TMS) activate or inhibit specific brain areas, often in an attempt to modulate networks whose connectivity has been associated with better recovery^23–25^. These stimulation methods may improve outcomes for those with stroke by permitting the development personalized treatment protocols. Recent TMS modeling work has shown that the effect of regional stimulation on FC depends on the brain state at the time of stimulation^26–28^. Determining subject-specific metrics of recurring patterns of brain activity may prove to have clinical benefit in the timing and spatial targets of stimulation treatments^29^.

Furthermore, the discovery of the effect of the dominant hemisphere in this paper may aid in refining how treatments are individually tailored. In a recent paper, Hordacre et al.^30^ propose a personalized model and suggest targeting alternative motor networks with tDCS. Based on this paper’s findings, we conjecture that this form of stimulation may only be appropriate in those with damage to the dominant hemisphere CST. Additionally, the direction of neural activity changes underlying FC differences across pathological groups compared to control populations has not yet been fully quantified. We showed that analyzing FC differences in the context of metrics of brain dynamics provides a more complete picture as to what activation patterns are driving observed differences. If the goal of stimulation therapies is to recapitulate functional network connectivity associated with better outcomes, then understanding how the activations within those networks give rise to connectivity differences may produce more effective targeting strategies.

There are several limitations to the study. First, the use of four clusters was chosen heuristically, and it is possible that more or fewer brain states exist in the stroke population. Second, k-means-based clustering of the time series does preserve the temporal resolution of brain states (as opposed to static or even dynamic FC), but with a few caveats. The true activation for a subject at a given time point is most similar to, but not identical to, the discrete cluster centroids described in this paper. There is significant variability in individual activation patterns which may be obscured by concatenating all subjects together and assigning each TR to a single, group-level cluster^11^. However, the goal of this study is to derive a meaningful group-level characterization of activity patterns after pontine stroke that may relate to motor recovery. Future work should address extending clustering results to individual subjects in order to support tailored treatments. Additionally, our fMRI sampling rate was 3 seconds which is too slow to capture faster, possibly relevant, brain dynamics^12,31^. Third, the frontoparietal network tends to be lateralized in motor-related activation; however, the clustering approach here uncovered a state with bilateral frontoparietal network activation. Therefore, determining whether contralesional or ipsilesional frontoparietal areas are more involved in recovery in subjects with dominant CST damage was not possible in this analysis. Fourth, the small sample size of subjects with dominant-hand CST damage limits confidence in the findings. Further analyses should attempt to replicate these findings with larger samples of subjects, including left-handed subjects with right hemisphere lesions. Additionally, CST integrity was assessed with the Dice coefficient and a population atlas which may not be accurate for all subject’s neuroanatomy. Finally, most of the individuals with dominant-hand CST damage in the model had left-hemisphere lesions, so we cannot fully rule out whether the changes are associated with the left hemisphere or the dominant hemisphere specifically. Similarly, this method is limited in its ability to distinguish the separate contribution of the ipsilateral and contralateral sensorimotor areas, which are known to be differentially activated after stroke^32^, as it seeks to determine recurring states present in healthy controls and stroke subjects. As a result, we expect that our results reflect adaptations/disruptions to “canonical” brain activation states present across stroke and control populations (and may change after stroke in the proportion or frequency with which they are expressed). Further work that investigates more states, possibly states specific to the post-CST stroke brain, may elucidate the role of contralesional hemisphere activation, but that is beyond the scope of this study.

## Methods

### Data description

The data consist of 23 first-episode stroke patients (34–74 years old; mean age 57 years; 8 female) with isolated pontine infarcts and 24 healthy sex-matched controls (33–65 years old; mean age 52 years; 10 female). Written informed consent was obtained from all participants. A subset of the data (11 stroke subjects and 11 healthy control subjects) used here has been previously described in ref. ^33^; the current study includes an additional 12 stroke subjects and 13 control subjects. Of the twenty-three stroke subjects, fourteen had right brainstem infarcts and nine had left brainstem infarcts (Supplementary Figs. 1a, 2, 3). Patients were scanned between two and five times over a period of 6 months. Specifically, MRIs were obtained at 7, 14, 30, 90, and 180 days after stroke onset on a 3T TimTrio Siemens using a 12-channel phase-array head coil. Fugl-Meyer assessments were performed twice for each subject at each session and averaged (Supplementary Fig. 1b). The Fugl–Meyer (FM) test includes 33 tasks that assesses motor function, balance, sensation, and joint function of the upper limbs^34^. Each task was rated on a scale of 0–2 (0 indicates the subject was unable to perform the task, 1 indicates the subject could partially perform the task, and two indicates the subject was able to perform the task). The total sum of the 33 scores was then normalized to a score between 0 and 100, where 100 represents the best possible performance across all 33 tasks. Anatomical images were acquired using a sagittal MP-RAGE three-dimensional T1-weighed sequence (TR, 1600 ms; TE 2.15 ms; flip angle, 9^∘^, 1.0 mm isotropic voxels, FOV 256 × 256). Each MRI session involved between two and four runs of task-free fMRI at 6 minutes each. Subjects were instructed to stay awake with their eyes open; no other task instruction was provided. Images were acquired using the gradient-echo echo-planar pulse sequence (TR, 3000 ms; TE, 30 ms; flip angle, 90^∘^, 3 mm isotropic voxels). Anatomical MRI, lesion masks, and fMRI data were processed as described below and in ref. ^35^.

### Anatomical MRI processing

Preprocessing of the longitudinal anatomical MRIs included affine registration of each subject’s T1 scans to the baseline T1 scan, collapsing co-registered files to an average T1, and creation of a skull-stripped brain mask followed by manual editing and binarization of the hand-edited mask. The brain mask was then transformed back to each of the follow-up T1s in native space using the inverse registration acquired from the first step. This was followed by bias field correction of all the T1 scans, transformation of native-space bias field-corrected data back to baseline space, and the creation of an average bias field-corrected scan for each subject. Stroke lesion masks were hand-drawn on these transformed T1 scans by ADB and JEB. Structural normalization was performed with the ANTs toolbox^36^.

### Functional MRI processing

Preprocessing of the longitudinal functional MRIs was performed using the CONN toolbox^37^, including functional realignment of volumes to the baseline volume, slice timing correction for alternating acquisition, segmentation, and normalization, and smoothing with a 4 mm FWHM kernel. This was followed by a denoising protocol (CompCor)^38^, which regressed out the cerebrospinal fluid and white matter signal, as well as 24 realignment parameters (added first-order derivatives and quadratic effects). Temporal band-pass filtering (0.008–0.09 Hz), despiking, and global signal removal regression were also performed. The first four frames of each BOLD run were removed. Frame censoring was applied to scans with a framewise displacement threshold of 0.5 mm along with its preceding scan^39^. Regional time series were acquired by parcellating the scans into 268 non-overlapping brain regions using a functional atlas derived from healthy controls^40^ and averaging the time course of all voxels within a given region. Voxels identified as lesioned were excluded from regional time series calculations. The first 200 volumes from each subject’s fMRI were used for subsequent analyses to ensure equal contribution of each scan to the brain state clustering (see below for details). Finally, each of 268 regions was assigned to one of eight functional networks, identified by ref. ^41^ using spectral clustering in healthy subjects (Supplementary Fig. 4) named as follows: medial frontal network, frontoparietal network, default mode network, subcortical/cerebellum network, motor network, visual I network, visual II network, and the visual association network. These networks reflect collections of brain regions whose temporal signals are homogeneous at rest (i.e., the activity of regions within each network is similar over time) in a healthy population, and are referred to as canonical networks due to their repeated observation in resting-state data.

### Dynamic brain states and their metrics

Following Cornblath et al., all subjects’ regional fMRI time series were concatenated, producing an *n* × *p* matrix where *n* = 47 subjects × 200 TRs × 2–5 sessions and *p* = 268 brain regions). This matrix was *z* scored along columns such that each brain region had a mean of 0 and a standard deviation of 1. *K*-means clustering was then applied to identify clusters of brain activation patterns, or states (Fig. 1a). Pearson correlation was used as the cluster distance metric and clustering was repeated 50 times with different random initializations before choosing the solution with the best separation of the data (minimum sum of point-to-centroid distances, summed over all k clusters). To determine the optimal number of clusters and evaluate the quality of clustering, we performed several analyses (Supplementary Fig. 5). First, we plotted the variance explained by clustering (between-cluster variance divided by the sum of between-cluster and within-cluster variance) for *k* = 2–12 and identified the curve’s elbow at *k* = 4 as a potential optimal number of clusters. We also plotted the distortion curve, which is the average distance from each point to its centroid and again determined via elbow criteria an optimal cluster number of 4. We then plotted silhouette coefficients for *k* = 4 to assess if there was evidence of misassignment of any of the points. To further assess the stability of clustering and ensure our partitions were reliable at *k* = 4, we repeated the above clustering process 50 times and compared the adjusted mutual information (AMI) between each of the 50 results. The partition which shared the greatest total AMI with all other partitions was selected as the final cluster assignment. The centroids of each state (cluster) were calculated by taking the mean of all TRs assigned to that state in regional activation space^6^. Following Cornblath et al., dominant networks in each state were determined by calculating the cosine similarity between each of eight networks and each centroid. High and low amplitude network-level activations were assessed separately by taking the cosine similarity of the positive and negative parts of the centroid (and zeroing out values with the opposite sign), respectively.

We performed several analyses to assess the robustness of our results under different conditions. First, we repeated the entire clustering process using two other brain atlases of varying resolutions: the group average FreeSurfer Desikan-Killany atlas with additional cerebellum and subcortical regions (86 regions)^42^ and the CC400 atlas (400 regions)^43^. Finally, we performed the clustering after combining stroke and control data together because we were interested in determining differences in shared activation states across both groups. However, it may be possible that stroke and control subjects occupy distinct states that can only be observed by clustering stroke and control subjects separately. Therefore, we repeated the clustering on the stroke and control subject data separately to determine if there were any differences in the resulting states compared to those obtained with the combined data.

FO, DT, AR, and TPs were calculated separately for each of the five sessions (1 week, 2 weeks, 1 month, 3 months, and 6 months post baseline or stroke) and the average FO/DT/AR/TPs for each subject was obtained by taking the mean across their longitudinal sessions. State dynamics metrics were compared between groups using unpaired two-tailed *t* tests and corrected for multiple comparisons using Benjamini–Hochberg (BH) and a false discovery rate (FDR) of 0.05^44^.

### Assessment of CST integrity

The probabilistic Tang brainstem atlas^45^ was used to define left and right binary CST masks in 2 mm MNI space by voxel-wise thresholding at 50%. The Dice overlap between the left and right CST masks and the binarized lesion mask was calculated for each subject and lesions were visualized to verify intersection with the CST (Supplementary Figs. 2 and 3). In one subject (SUB13), Dice overlap with CSTs were low; however, upon visualization the lesion appeared to impact CST ventral to the atlas, i.e., in the spinal cord. This was confirmed by assessing the lesion’s overlap with a spinal cord atlas (Supplementary Fig. 12) using the Spinal Cord Toolbox^46^. In total, 21/23 subjects had CST damage and 10/23 subjects had damage to their dominant-hand CST, but only 9/23 had motor assessment data at the 3- and/or 6-month time points. Of the nine subjects with dominant CST damage and motor scores, 7/23 were right-handed and either had left CST damage superior to decussation or right spinal cord CST damage inferior to decussation, 1/23 was left-handed with bilateral damage, and 1/23 was left-handed with right CST damage (Supplementary Table 1).

### Relating *FPN*^+^ state metrics to chronic motor outcomes

We were interested in determining whether *FPN*^+^ state metrics related to longer-term (chronic) motor performance outcomes specifically in subjects with dominant hemisphere CST damage. To assess the interaction effect of hand dominance on the relationship between the frontoparietal state parameters (FO and DT) over time and FM scores over time, two linear models were constructed:

1 $$\Delta F{M}_{{{{{\rm{chronic}}}}}} \sim CS{T}_{D}+\Delta D{T}_{{{{{\rm{chronic}}}}}}^{FP{N}^{+}}+CS{T}_{D}* \Delta D{T}_{{{{{\rm{chronic}}}}}}^{FP{N}^{+}}+\beta$$

2 $$\Delta F{M}_{{{{{\rm{chronic}}}}}} \sim CS{T}_{D}+\Delta F{O}_{{{{{\rm{chronic}}}}}}^{FP{N}^{+}}+CS{T}_{D}* \Delta F{O}_{{{{{\rm{chronic}}}}}}^{FP{N}^{+}}+\beta$$

where:

3 $$\Delta F{M}_{{{{{\rm{chronic}}}}}}=F{M}_{{{{{\rm{chronic}}}}}}-F{M}_{{{{{\rm{baseline}}}}}}$$

4 $$\Delta D{T}_{{{{{\rm{chronic}}}}}}^{FP{N}^{+}}=D{T}_{{{{{\rm{chronic}}}}}}^{FP{N}^{+}}-D{T}_{{{{{\rm{baseline}}}}}}^{FP{N}^{+}}$$

5 $$\Delta F{O}_{{{{{\rm{chronic}}}}}}^{FP{N}^{+}}=F{O}_{{{{{\rm{chronic}}}}}}^{FP{N}^{+}}-F{O}_{{{{{\rm{baseline}}}}}}^{FP{N}^{+}}$$

and *CST*_*D*_ is a binary variable indicating whether the subject’s dominant-hand CST had non-zero Dice overlap with the lesion. *FM*_baseline_ was set to the earliest *FM* score available for each subject, which was the FM score at 1-week post-stroke for most of the subjects. For two subjects (SUB22 and SUB23), 1 week FM scores were not available; the baseline FM scores for these subjects was estimated by their oldest FM score (2 weeks and 1 month, respectively). *FM*_chronic_ was set to the most recent chronic FM score (i.e., at 3 or 6 months after stroke, whichever is later). Only one subject (SUB6) had a FM score at 3 months post-stroke but not 6 months post stroke. Two subjects (SUB12 and SUB20) were excluded from the model entirely as they did not have any FM scores or imaging data in their chronic time period, beyond 1 month and 2 weeks, respectively. The models were constructed using only the *FPN*^+^ metrics that were found to have significant differences between stroke and controls at the 3 and 6-month time points. *P*-values obtained for each predictor across all models were corrected for multiple comparisons using BH-FDR and a threshold of 0.05.

### Effect of proportional recovery on observed relationships

We were interested in determining whether relationships observed in the above model were driven by the correlation (if any) between DT/FO in *FPN*^+^ and baseline FM scores in subjects with dominant-hand CST damage. Because most subjects obtained proportional recovery or greater, i.e. their final impairment was ≥70% of their initial impairment^47^, their 1-week FM scores were strongly correlated with 3- and 6-month FM scores (a phenomenon called the ceiling effect, see ref. ^48^). Therefore, it is possible that relationships between Δ*FM* and $\Delta FO/D{T}^{FP{N}^{+}}$ observed in the linear model could be driven by an underlying relationship between baseline FM scores and $\Delta FO/D{T}^{FP{N}^{+}}$. To explore this possibility, for those *FPN*^+^ metrics whose change had a significant correlation with baseline FM (which were chronic $\Delta D{T}^{FP{N}^{+}}$ and $\Delta F{O}^{FP{N}^{+}}$), we performed permutation testing to obtain the null distribution of correlations between Δ*FM* and $\Delta FO/D{T}^{FP{N}^{+}}$, assuming patients obtain only proportional recovery (PR). *FM*_baseline_, $FO/D{T}_{{{{{\rm{baseline}}}}}}^{FP{N}^{+}}$ and $FO/D{T}_{{{{{\rm{chronic}}}}}}^{FP{N}^{+}}$ were fixed to their actual observed values, but $F{M}_{{{{{\rm{chronic}}}}}}^{PR}$ was randomly generated by simulating scores according to the proportional recovery rule as in ref. ^47^. Specifically, each subject’s FM scores ($F{M}_{{{{{\rm{chronic}}}}}}^{PR}$) were set to 70% of their initial impairment (100-*FM*_baseline_) with an noise term *ϵ* ~ *N*(0, 3):

6 $$F{M}_{{{{{\rm{chronic}}}}}}^{PR}=0.7* \left(100-{FM}_{{{{{\rm{baseline}}}}}}\right)+ \epsilon ,$$

The *p*-value for the correlation between the observed Δ*FM* and $\Delta FO/D{T}^{FP{N}^{+}}$ and was then calculated by calculating the proportion of times the null correlation exceeded the true correlation (Supplementary Fig. 14c, d). If that *p*-value is significant, then we can be more confident that $\Delta FO/D{T}^{FP{N}^{+}}$ is correlated with the change in FM above and beyond baseline FM and the expected proportional recovery.

### Comparison of FO and FC

Finally, we wanted to understand how differences in state dynamics between controls and stroke subjects could translate to differences in FC between the two groups. We hypothesize that individuals with more TRs (higher FO) in a brain state with high co-activation of two networks will result in larger positive FC between those networks, while more TRs (higher FO) in a brain state with large activations in opposite directions (contra-activation) of two networks will result in a more negative FC between those networks (Supplementary Fig. 15). We tested this hypothesis for our brain state of interest, *FPN*^+^, in the following way. First, we identified the pairs of networks that were highly co-activated/contra-activated during *FPN*^+^, which was defined as having an absolute value cosine similarity with the centroid of *FPN*^+^ of greater than 0.2 (chosen heuristically as the threshold separating networks active vs. not active during a given state). We only analyzed the networks with larger magnitude co-activations/contra-activations since the networks with activity closer to zero in the *FPN*^+^ state are not likely to be influenced by changes in FO of this state. We first calculated the FC as the Pearson’s correlation between each pair of 268 regions and performed a Fisher’s r-to-z transformation of the FC weights. We then averaged the FC values between regions belonging to each pair of networks to produce a network-level FC (i.e., the average FC within each of 8 predefined networks, including the frontoparietal network). We correlated each subject’s FO in the *FPN*^+^ state with the FC between each pair of networks determined to be highly co-activated/contra-activated in the *FPN*^+^ state. In an analysis inspired by ref. ^12^, we further demonstrated that temporal fluctuations in $F{O}^{FP{N}^{+}}$ over segments of the scan are related to sliding-window FC in nodes of the frontoparietal network. We first identified regions with high z-score BOLD signal in the *FPN*^+^ centroid (>0.4), and, for those regions, calculated their sliding-window FC and $F{O}^{FP{N}^{+}}$ using the same window and overlap (window size = 45 seconds, overlap = 3 seconds). We then correlated these two values over the entire fMRI scan for each individual. To ensure any observed relationship was not driven by global BOLD fluctuations, we recalculated this correlation using 100 randomly selected regions’ dynamic FC as a null comparison.

#### Statistics and reproducibility

We calculated statistics comparing brain dynamic parameters between stroke and control groups. Where stated, comparisons were corrected for multiple comparisons to reduce type I error. The clustering was repeated 50 separate times to ensure the final solution was not in large disagreement with other possible solutions. We replicated the main analyses with *k* = 5 brain states and found a general agreement between the results obtained with *k* = 4. Code for the analyses in this manuscript have been made publicly available.

### Reporting summary

Further information on research design is available in the Nature Research Reporting Summary linked to this article.

## Supplementary information

Supplementary Information

Description of Additional Supplementary Files

Supplementary Data 1

Supplementary Data 2

Reporting Summary

## Data Availability

Data are not publicly available due to privacy issues regarding clinical data. Raw data to generate Figs. 3 and 6e can be found in the file Supplementary Data 1 and 2, respectively. All other data can be made available upon request to the corresponding authors on the condition that a formal data sharing agreement is made.

## Code availability

The code to replicate this analysis is available on GitHub: https://github.com/emilyolafson/dynamic-brainstates and Zenodo^49^.
