# Supplementary material for: Frontoparietal network activation is associated with motor recovery in ischemic stroke patients
Source: Commun Biol. 2022 Sep 21;5:993. doi: 10.1038/s42003-022-03950-4 (PMC9492673; doi:10.1038/s42003-022-03950-4)
Supplement: Supplementary file 1 — Supplementary Information [file 42003_2022_3950_MOESM1_ESM.pdf]

Supplementary Figures

ID	Lesion Location	Handedness	Dominant CST damage
SUB1	Right Pons	R	Non-dominant CST
SUB2	Right Pons	R	Non-dominant CST
SUB3	Left Pons	R	Dominant CST
SUB4	Left Pons	R	Dominant CST
SUB5	Right Pons	R	Non-dominant CST
SUB6	Left Pons and midbrain*	L	Dominant CST
SUB7	Right Pons	L	Dominant CST
SUB8	Right Pons	R	Non-dominant CST
SUB9	Right Pons	R	Non-dominant CST
SUB10	Right Pons	R	Non-dominant CST
SUB11	Left Pons	R	Dominant CST
SUB12	Left bulbus medullae	B	Dominant CST
SUB13	Left bulbus medullae	R	Dominant CST
SUB14	Right bulbus medullae	R	Non-dominant CST
SUB15	Right Pons and cerebellum	R	Non-dominant CST
SUB16	Left Pons	R	Dominant CST
SUB17	Left Pons	R	Dominant CST
SUB18	Right Pons	R	Non-dominant CST
SUB19	Right Pons	R	Non-dominant CST
SUB20	Right Pons	R	Non-dominant CST
SUB21	Right Pons	R	Non-dominant CST
SUB22	Left Pons	R	Dominant CST
SUB23	Right Pons	R	Non-dominant CST

Supplementary Table 1: Breakdown of subjects according to lesion side, handedness, and dominant-hemisphere CST damage. Blue subjects indicate those that were included in the linear model as having "Dominant CST damage" assessing the effect of CST damage on FPN+ recruitment in recovery. Note that SUB12 has damage to their dominant CST but does not have motor scores for the chronic period, and was not included in the model. L; left, R; right, B; both. *indicates lesion overlaps with L and R CST tracts.

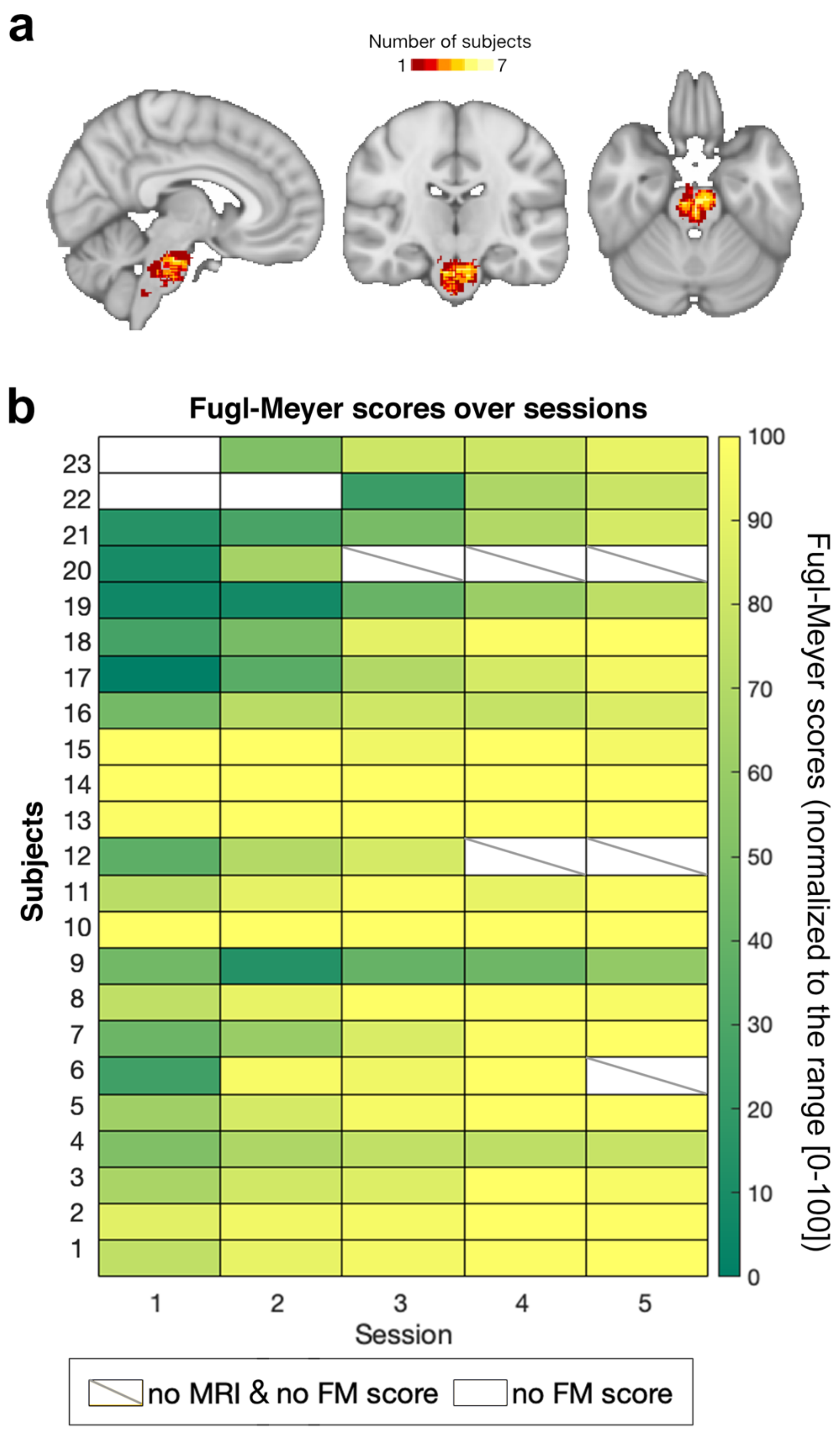

Supplementary Figure 1: Lesion and motor scores for pontine stroke subjects. **a**. Distribution of lesions. **b**. Fugl-Meyer scores (normalized 0-100).

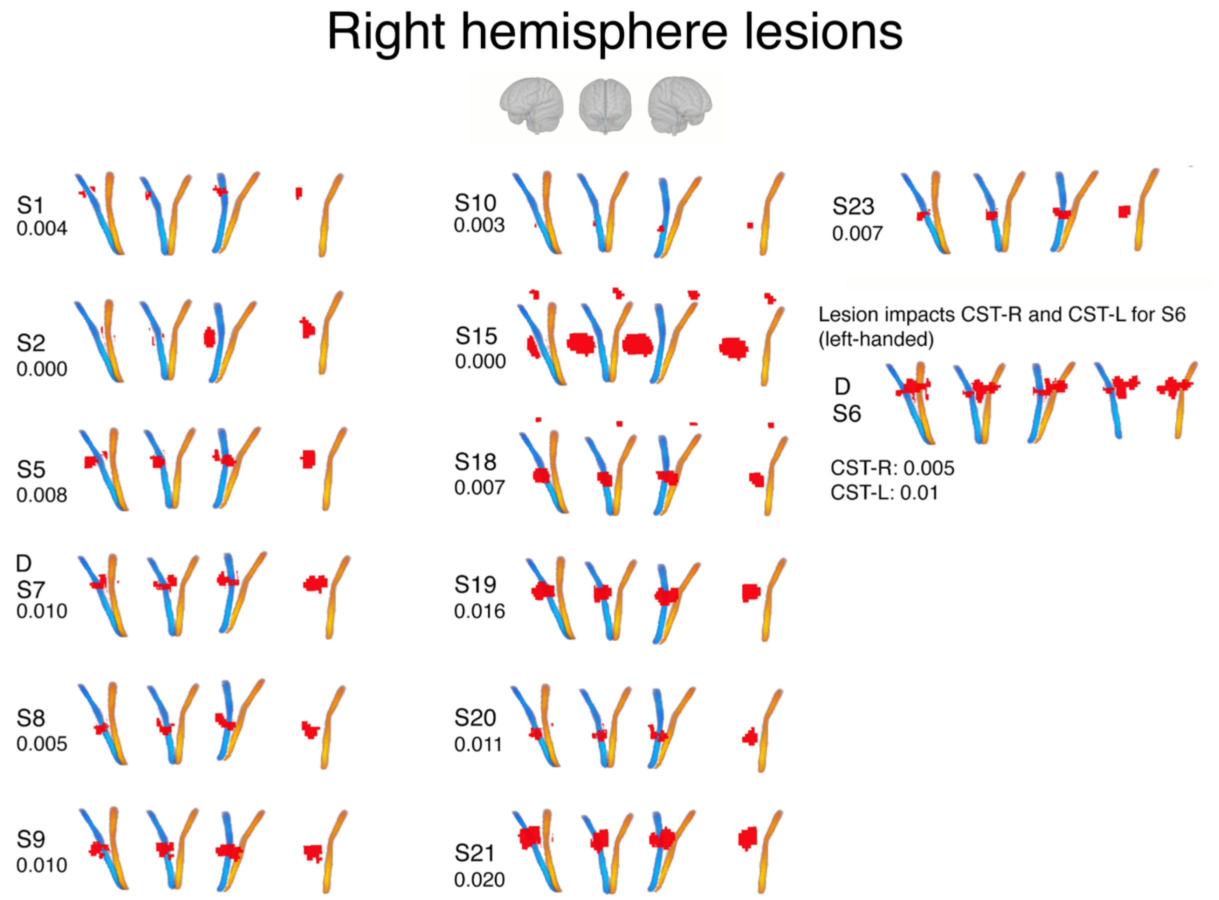

Supplementary Figure 2: Right hemisphere lesion location relative to brainstem corticospinal tracts. Red = lesion. Blue = right CST, yellow = left CST. Four views of the lesions/CSTs are displayed, from left to right: left lateral view, anterior view, right lateral view. A "D" above the subject identifiers ("SX") indicates that the lesion is in that subject's dominant hemisphere. Numbers below subject identifiers indicate Dice overlap between lesion and ipsilesional CST.

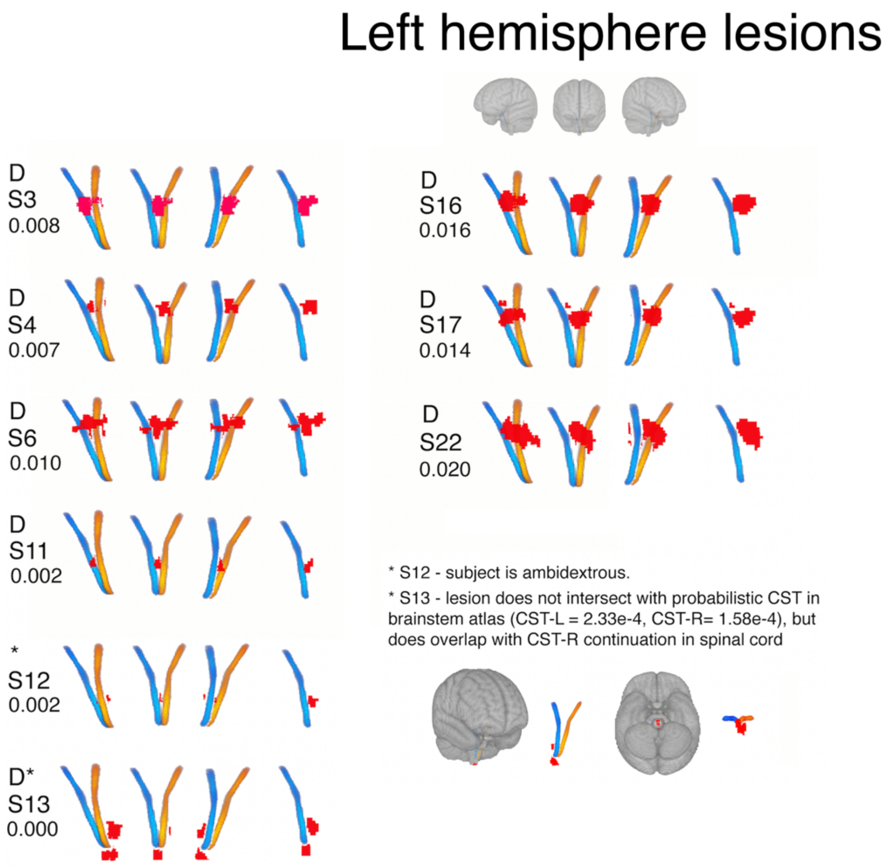

Supplementary Figure 3: Left hemisphere lesion location relative to brainstem corticospinal tracts. Red = lesion. Blue = right CST, yellow = left CST. Four views of the lesions/CSTs are displayed, from left to right: left lateral view, anterior view, right lateral view. A "D" above the subject identifiers ("SX") indicates that the lesion is in that subject's dominant hemisphere. Numbers below subject identifiers indicate Dice overlap between lesion and ipsilesional CST.

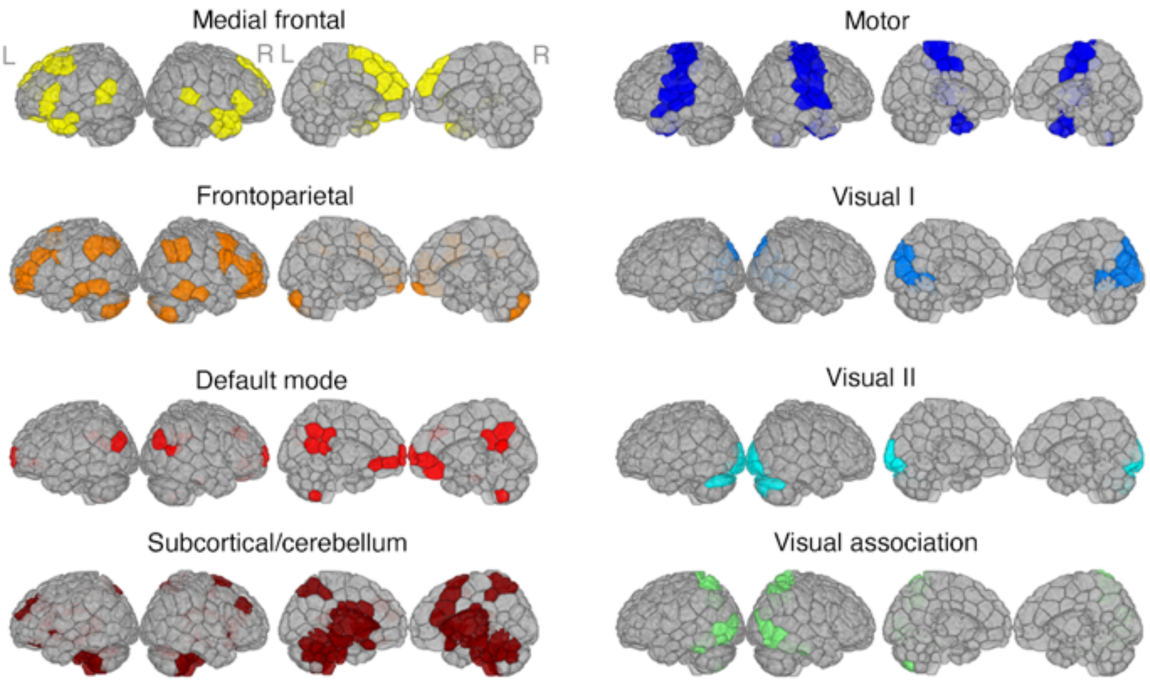

Supplementary Figure 4: Functional networks used in analysis (based on Shen et al., 2013) that represent communities of brain regions with similar activity over time. Colors are random to distinguish different networks.

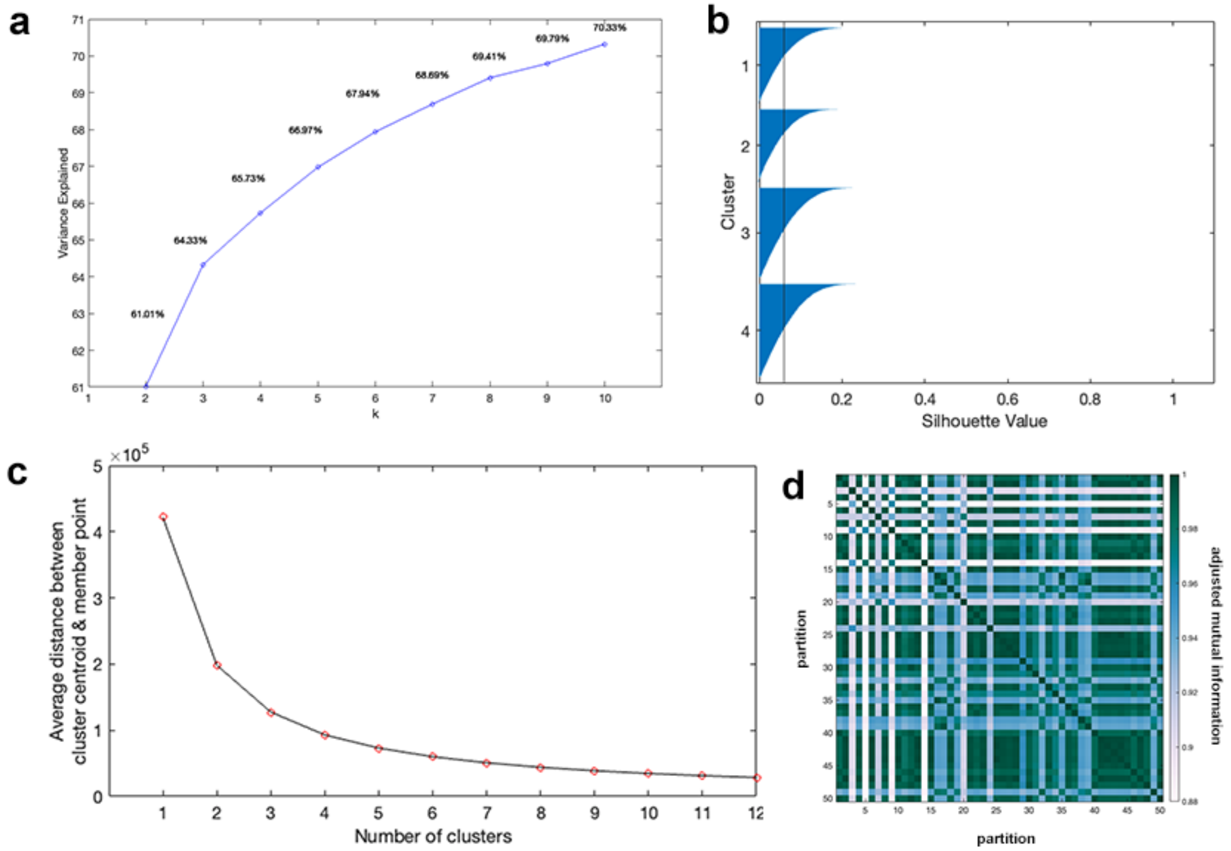

Supplementary Figure 5: Clustering criteria used to determine the optimal cluster number. **a.** Elbow plot: the total variance explained by increasing k from k=3 to k = 4 is 1.4%; from k = 4 to k = 5 is 1.2%. **b**. Silhouette values for k = 4 where negative values indicate that a data point (TR) may have been assigned to the wrong cluster. **c**. Average distance between cluster centroids and each member point (distortion criteria). **d**. Adjusted mutual information between 50 final clusters with varied initialization of k-means when k is set to 4 (minimum value observed = 0.88).

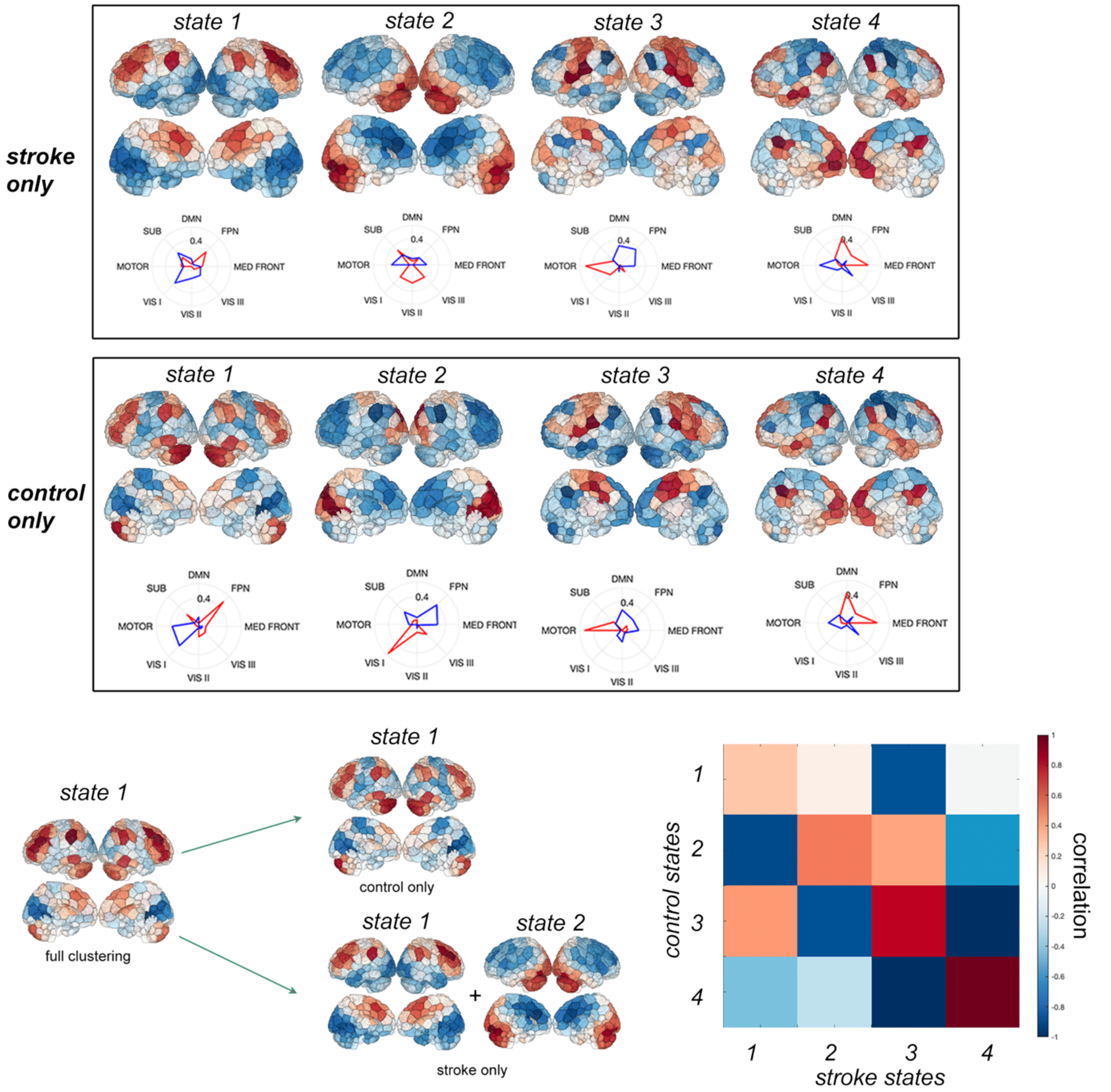

Supplementary Figure 6: Centroids derived from clustering stroke and control subjects separately. Bottom left: Hypothesized mapping from the centroids derived from clustering both groups together to the individually clustered centroids. Bottom right: Correlation of the centroids derived from separately clustering control and stroke groups

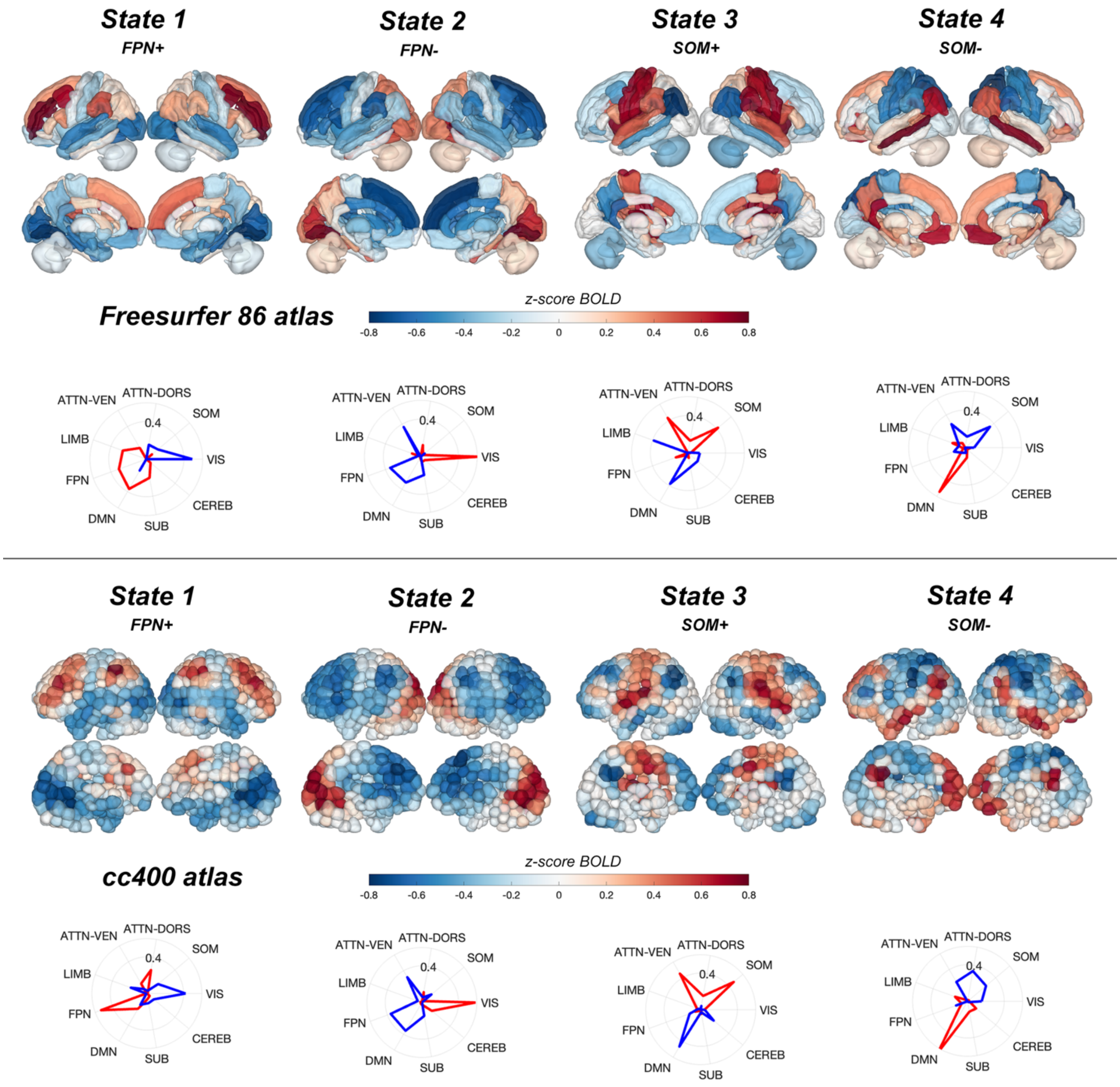

Supplementary Figure 7: Centroids derived from clustering fMRI data parcellated using different atlases: (top) FreeSurfer-86 region (group-average; not individual anatomical parcellations) and (bottom) CC400. Note that the regions are parcellated into 9 networks here, instead of 8, and there are missing (VIS II, VIS III, MED FRONT) and additional (ATTN-VEN, ATTN-DORS, LIMB) networks that are not in the shen268 network parcellation. SUB + CEREB are also combined in the shen268 parcellation, and the label SOM is used here instead of MOTOR in the main results.

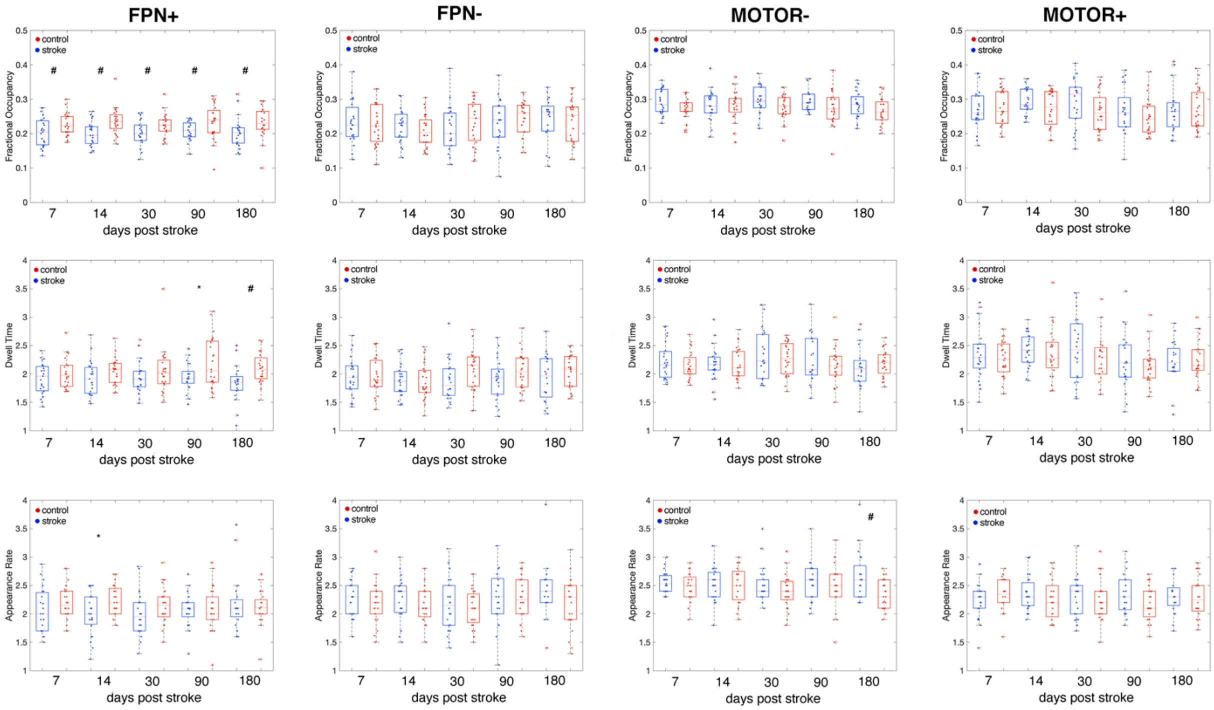

Supplementary Figure 8: Full session-specific group differences in fractional occupancy (top row), dwell time (middle row), and appearance rate (bottom row) with k = 4. Aside from the group differences reported in the main paper in FPN^{+}, there are group differences in $AR^{MOTOR+}{}_{6\ months.}$ Boxplot whiskers indicate the 25th and 75th percentiles, and the central mark indicates the median.

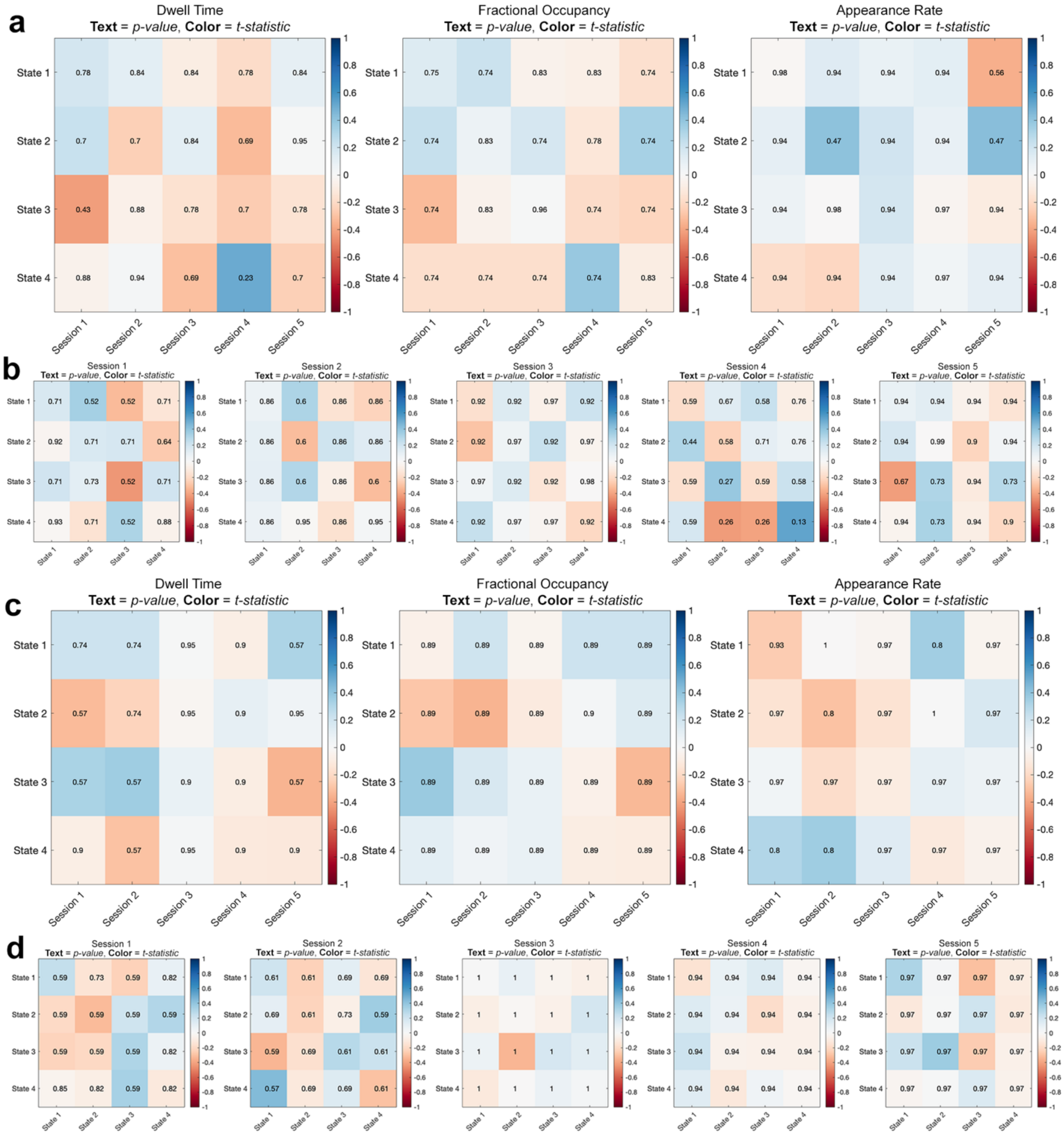

Supplementary Figure 9: Correlation between brain state parameters and age in control and stroke subjects. Colors indicate Pearson's correlation coefficient, text represents Benjamini-Hochberg corrected p-values (alpha = 0.05). **a**. Correlation between age and dwell time, fractional occupancy, and appearance rate in control subjects. **b**. Correlation between age and transition probabilities in control subjects. **c**. Correlation between age and dwell time, fractional occupancy, and appearance rate in stroke subjects. **d**. Correlation between age and transition probabilities in stroke subjects.

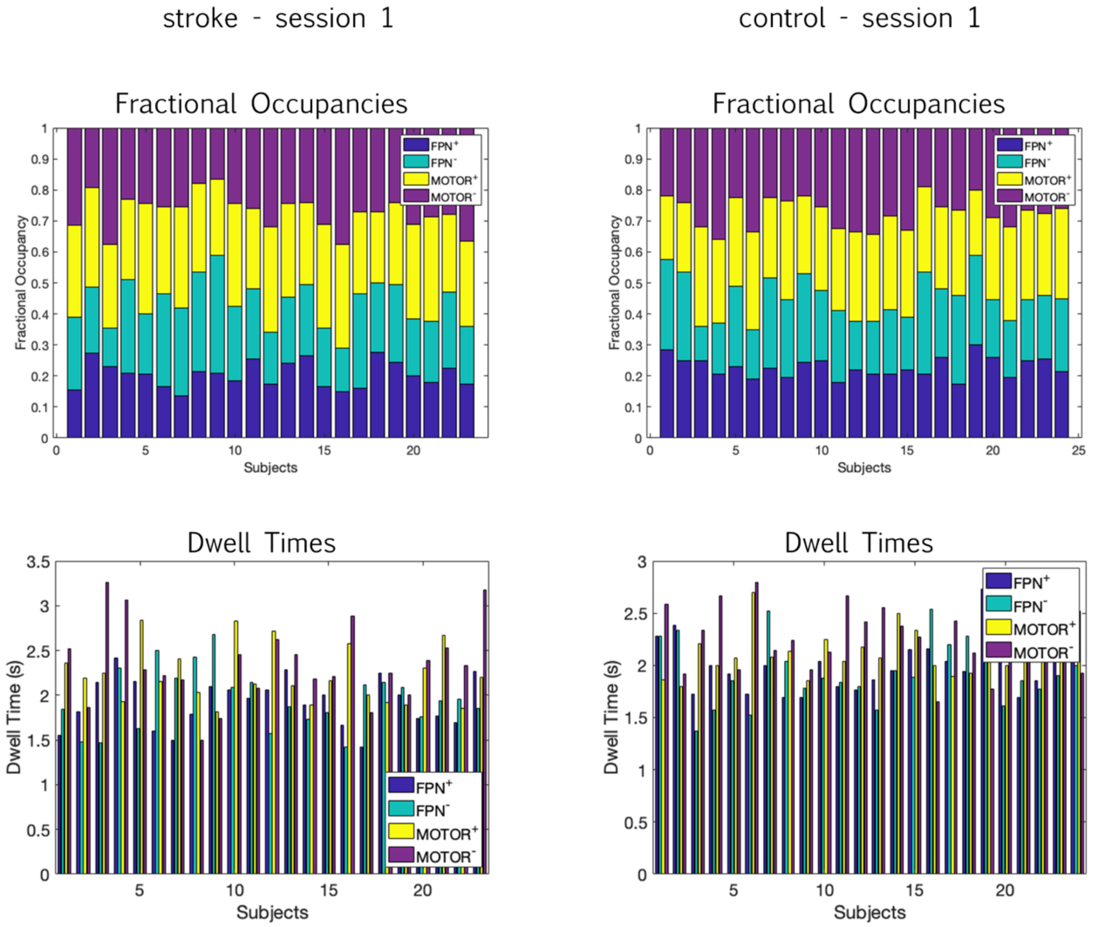

Supplementary Figure 10: Dwell times and fractional occupancies of stroke subjects (left) and control subjects (right) at session 1.

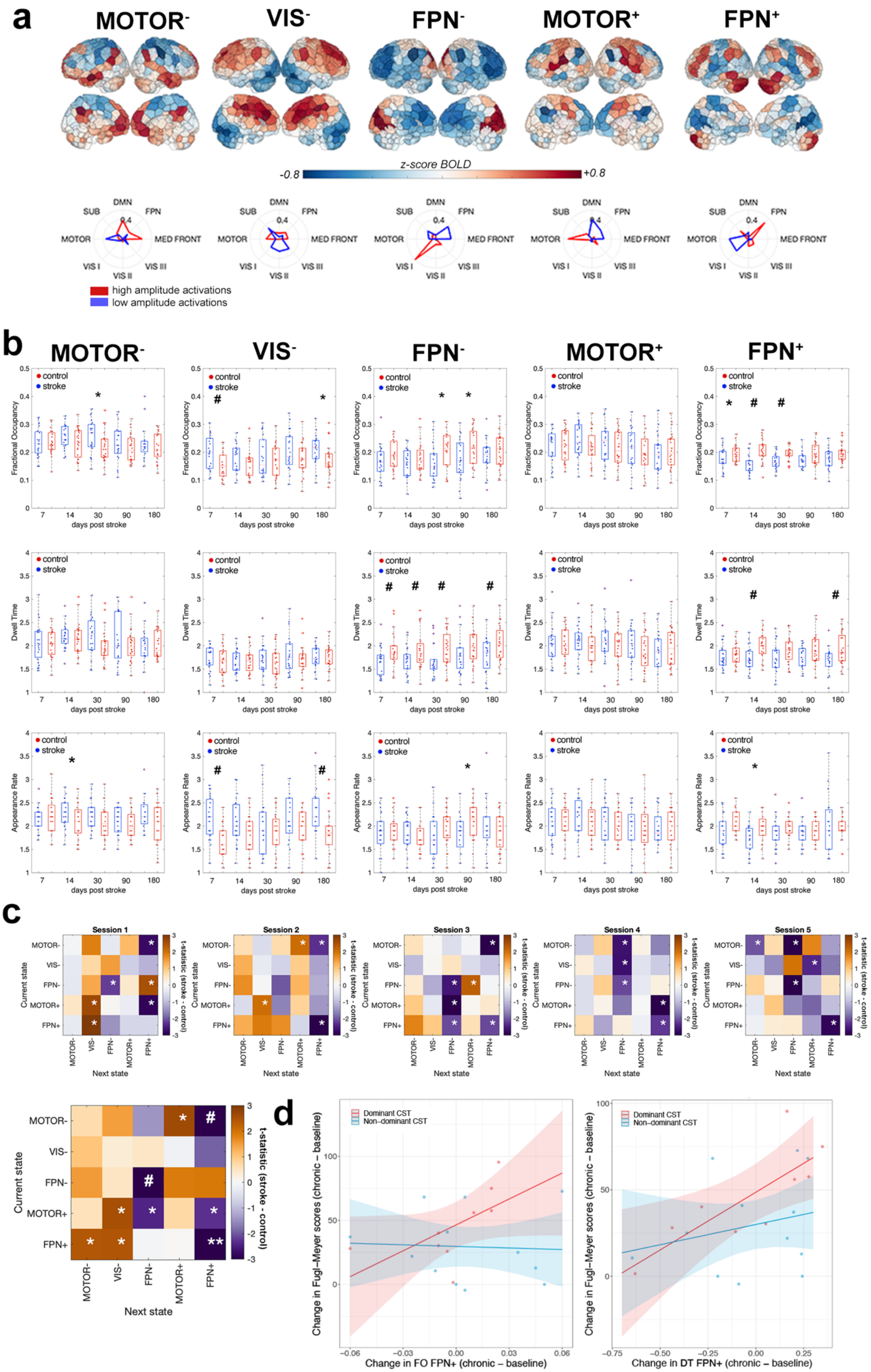
a
MOTOR-
VIS-
FPN-
MOTOR+
FPN+
z-score BOLD
-0.8
+0.8
DMN
FPN
MED FRONT
VIS III
VIS II
VIS I
MOTOR
SUB
high amplitude activations
low amplitude activations
b
Fractional Occupancy
Dwell Time
Appearance Rate
control
stroke
days post stroke
c
Session 1
Session 2
Session 3
Session 4
Session 5
Current state
Next state
t-statistic (stroke - control)
d
Dominant CST
Non-dominant CST
Change in Fugl-Meyer scores (chronic – baseline)
Change in FO FPN+ (chronic – baseline)
Change in DT FPN+ (chronic – baseline)

Supplementary Figure 11: Main results from the paper replicated with k = 5 clusters. **a**. Cluster centroids from k = 5 show that largely the same states appear (FPN^+, FPN^-, $MOTOR^+$, $MOTOR^-$) alongside a new state (VIS^-) characterized by low amplitude activations of the visual network. **b**. Stroke-control differences in fractional occupancy (top row), dwell time (middle row), and appearance rate (bottom row) mirror differences observed with k = 4, particularly group differences in FO and DT in FPN^+. Notably, FPN^- displays significant group differences in DT using k = 5 but not k = 4. Boxplot whiskers indicate the 25th and 75th percentiles, and the central mark indicates the median. **c**. Stroke-control differences in transition probabilities between states mirrors results with k = 4, particularly reduced transition probability from MOTOR into FPN^+ and reduced persistence probability of FPN^+. **d**. Linear model results examining the relationship between longitudinal changes in DT^{FPN+} and FO^{FPN+} and motor recovery in subjects with dominant hemisphere CST damage. Trend-level effects are replicated with k = 5 but do not reach statistical significance (p-value of marginal effect of $\Delta DT^{FPN+} = 0.0339$ (uncorrected), $\Delta FO^{FPN+} = 0.10640$ (uncorrected)). Interaction effects are not replicated.

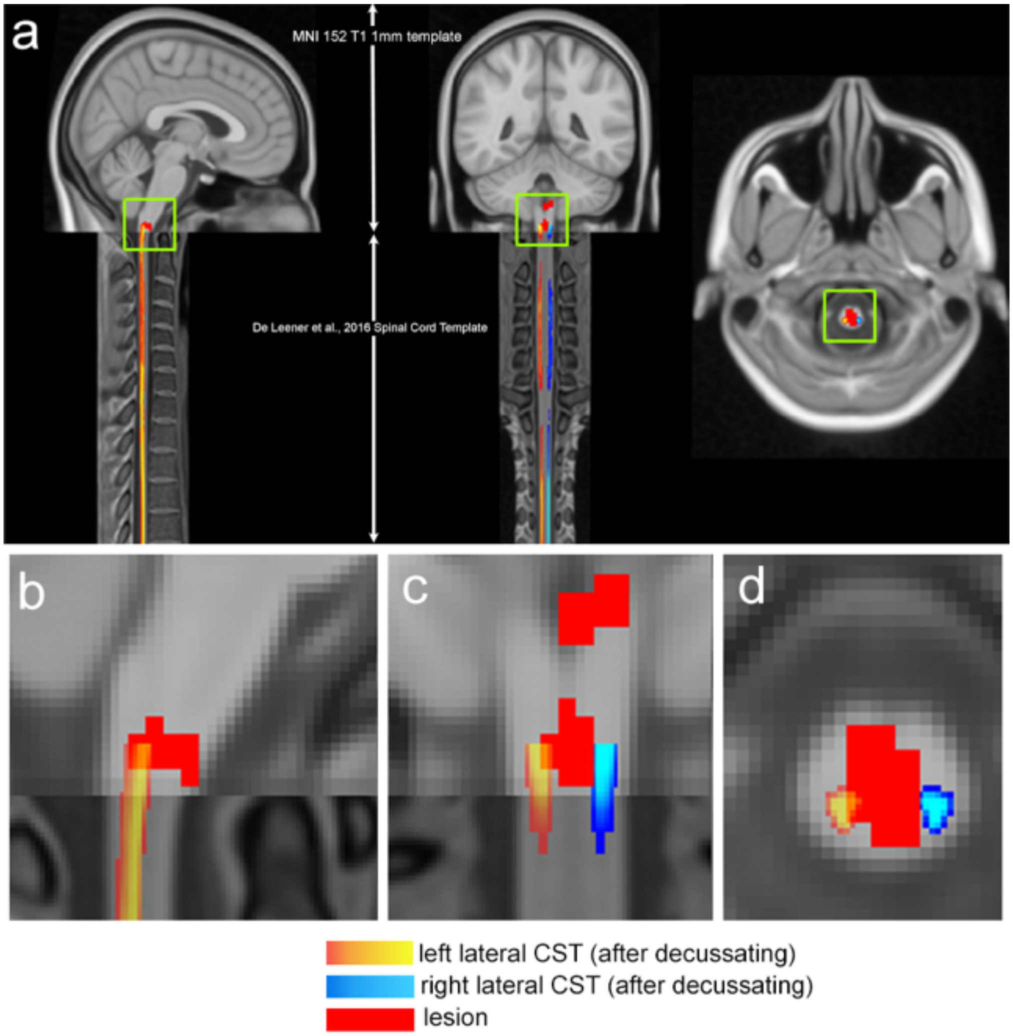

Supplementary Figure 12: Lesion for subject S13 alongside CST defined in a spinal cord atlas. **a**. Sagittal, coronal, and horizontal views of the lateral corticospinal tract atlases and lesion for subject S13 (who is right-handed) on top of the MNI 152 brain template (top) and De Leneer et al. spinal cord template. Close-ups of **b**., sagittal slice, **c**., coronal slice, and **d**., horizontal slice with reduced opacity of the left lateral CST, which is the dominant CST as the subject is right-handed and the lesion is in the right spinal cord after decussating in the medulla.

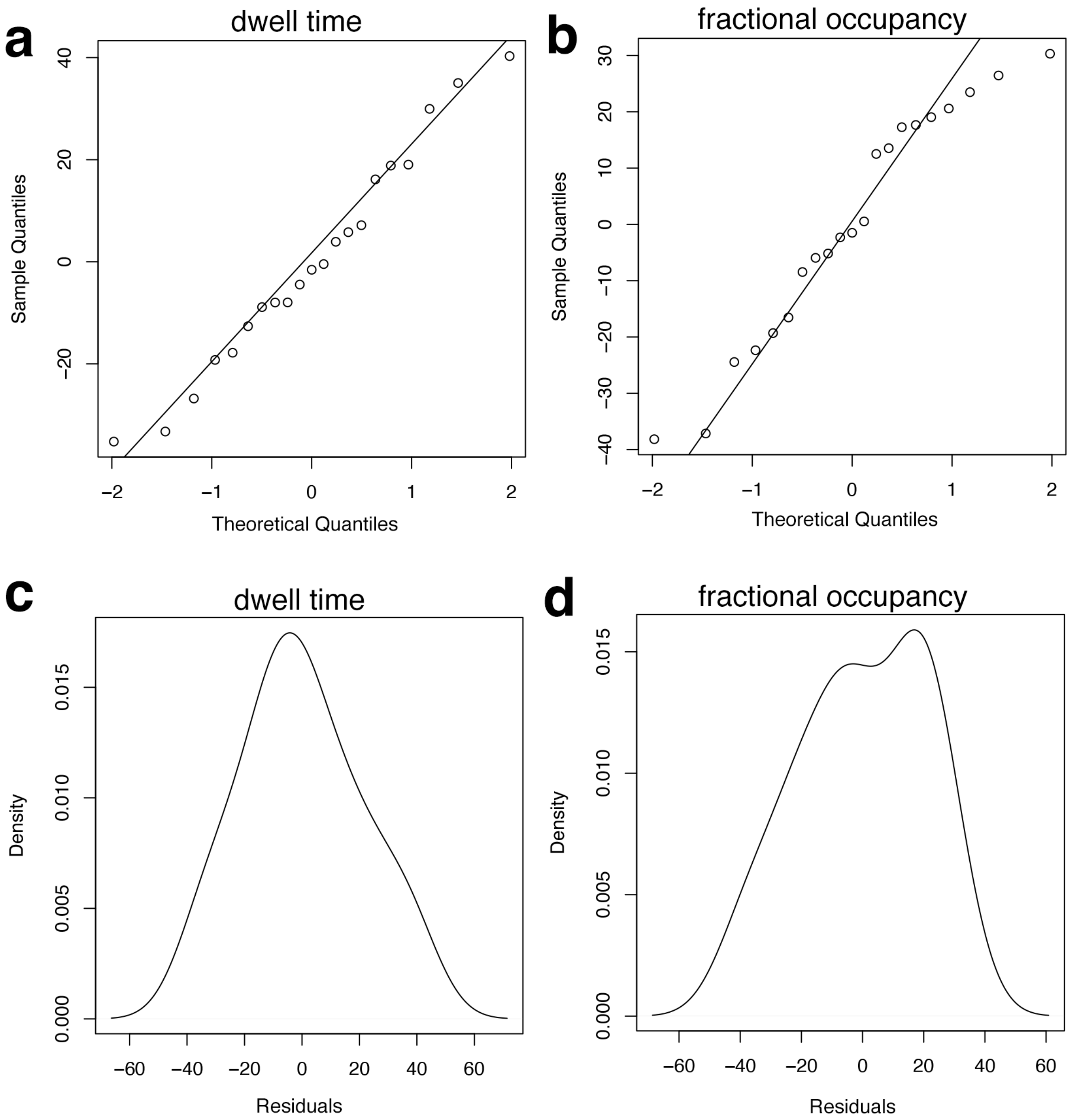

Supplementary Figure 13: Assessment of linear model normality assumption. **a, b.** QQ-plots comparing the empirical and theoretical quantiles of the linear models. **c, d.** histograms of residuals of the linear models.

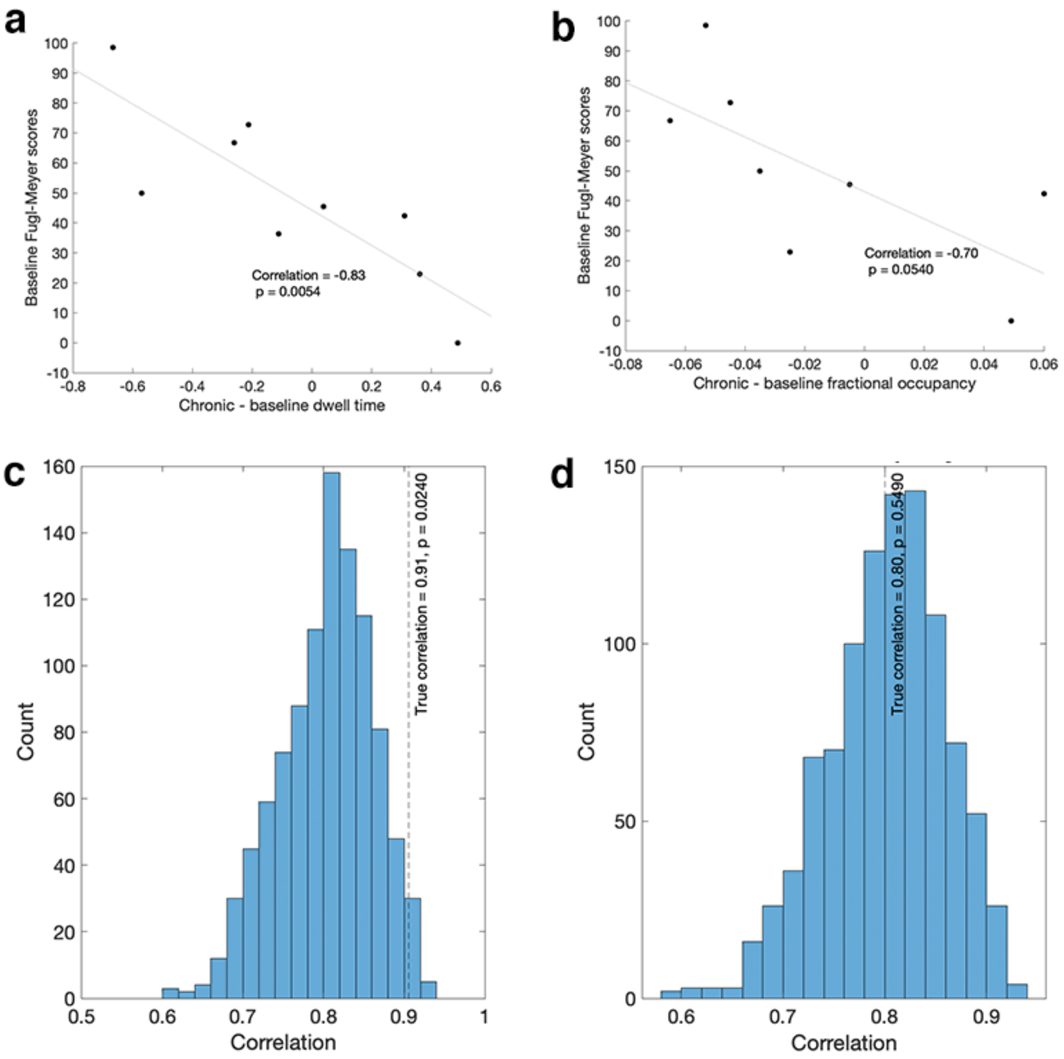

Supplementary Figure 14: Assessing the relationship between change in FPN+ state parameters and Fugl-Meyer scores in subjects with dominant hemisphere CST damage: is the association observed due to correlations between baseline Fugl-Meyer and chronic Fugl-Meyer scores? **a**. Correlation between baseline Fugl-Meyer scores and the change in dwell time in FPN^+ between baseline and chronic time points in subjects with dominant hemisphere CST damage. **b**. Correlation between baseline Fugl-Meyer scores, and the change in fractional occupancy in FPN^+ between baseline and chronic time points in subjects with dominant hemisphere CST damage. **c.** Distribution of correlations between change in dwell time and change in Fugl-Meyer using observed baseline and chronic dwell times, observed baseline Fugl-Meyer scores, and chronic Fugl-Meyer scores created according to the proportional recovery plus random noise (100 sets of chronic Fugl-Meyer scores were generated). Observed correlation using observed chronic Fugl-Meyer scores is shown as the grey dotted line. **d**. Distribution of correlations between change in fractional occupancy and change in Fugl-Meyer using observed baseline and chronic fractional occupancy, observed baseline Fugl-Meyer scores, and chronic Fugl-Meyer scores created according to the proportional recovery plus random noise (100 sets of chronic Fugl-Meyer scores were generated). Observed correlation using observed chronic Fugl-Meyer scores is shown as the grey dotted line.

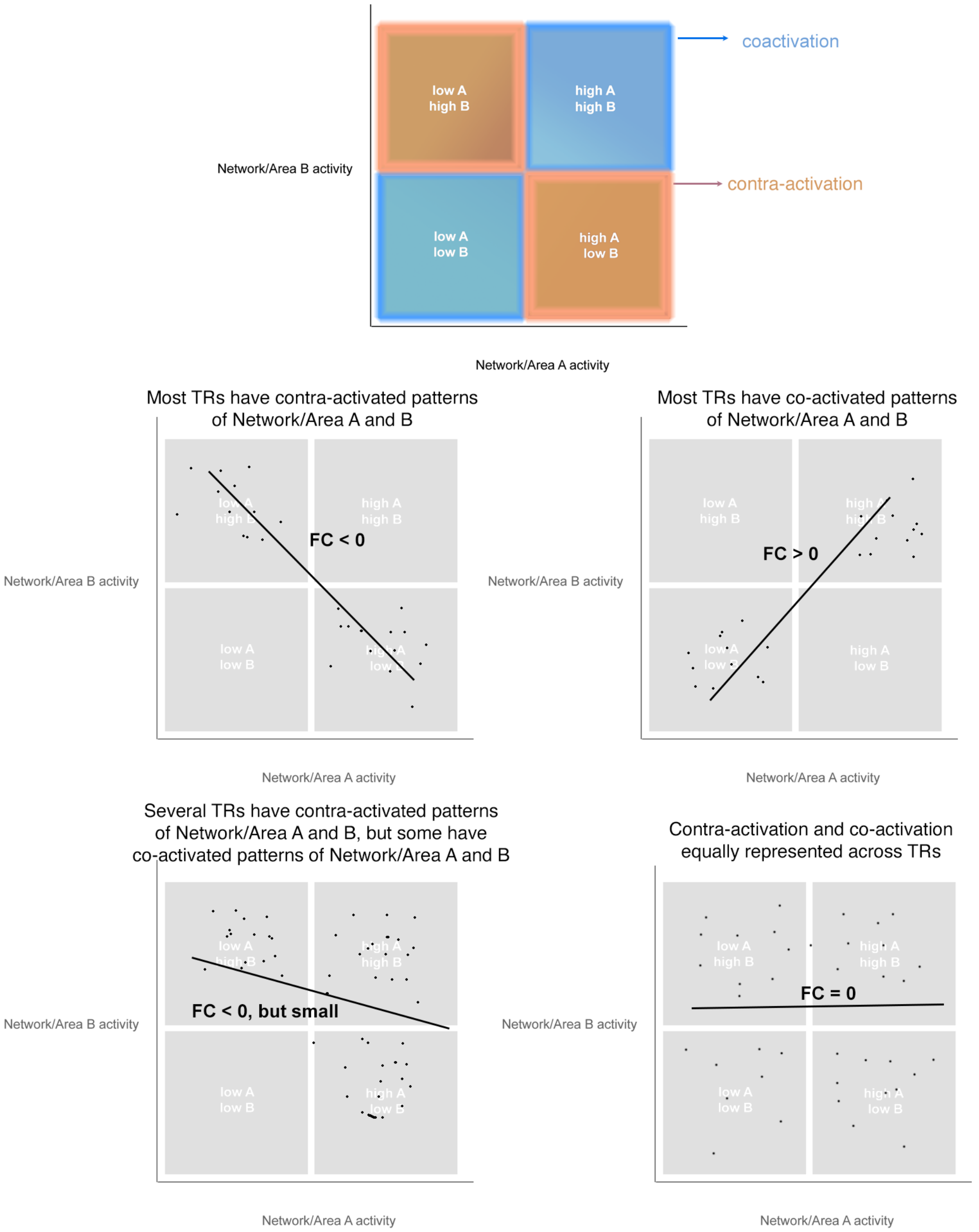

Supplementary Figure 15: Theoretical framework for linking fractional occupancy of brain states to functional connectivity between brain regions. Each TR can be plotted into one of four quadrants representing the joint activity patterns of a given pair of networks: high activity of networks/regions A and B (coactivation); low activity of A and B (coactivation); high A, low B (contra-activation); and low A, high B (contra-activation). When most TRs are in a contra-activation configuration of 2 areas, then the functional connectivity between those areas will be negative; when most TRs are in a co-activation configuration, the FC between those two areas will be positive.
