# Supplementary material for: Frontoparietal network activation is associated with motor recovery in ischemic stroke patients
Source: Commun Biol. 2022 Sep 21;5:993. doi: 10.1038/s42003-022-03950-4 (PMC9492673; doi:10.1038/s42003-022-03950-4)
Supplement: Supplementary file 2 — Description of Additional Supplementary Files [file 42003_2022_3950_MOESM2_ESM.pdf]

Description of Additional Supplementary Files

File name: Supplementary Data 1
Description: Raw data to generate Figure 3.

File name: Supplementary Data 2
Description: Raw data to generate Figure 6e.
