# Supplementary material for: Frontoparietal network activation is associated with motor recovery in ischemic stroke patients
Source: Commun Biol. 2022 Sep 21;5:993. doi: 10.1038/s42003-022-03950-4 (PMC9492673; doi:10.1038/s42003-022-03950-4)
Supplement: Supplementary file 5 — Reporting Summary [file 42003_2022_3950_MOESM5_ESM.pdf]

nature portfolio

Corresponding author(s): Emily Olafson

Last updated by author(s): May 17, 2022

Reporting Summary

Nature Portfolio wishes to improve the reproducibility of the work that we publish. This form provides structure for consistency and transparency in reporting. For further information on Nature Portfolio policies, see our Editorial Policies and the Editorial Policy Checklist.

Statistics

For all statistical analyses, confirm that the following items are present in the figure legend, table legend, main text, or Methods section.

n/a	Confirmed	
☐	☒	The exact sample size (n) for each experimental group/condition, given as a discrete number and unit of measurement
☐	☒	A statement on whether measurements were taken from distinct samples or whether the same sample was measured repeatedly
☐	☒	The statistical test(s) used AND whether they are one- or two-sided<br>*Only common tests should be described solely by name; describe more complex techniques in the Methods section.*
☐	☒	A description of all covariates tested
☐	☒	A description of any assumptions or corrections, such as tests of normality and adjustment for multiple comparisons
☐	☒	A full description of the statistical parameters including central tendency (e.g. means) or other basic estimates (e.g. regression coefficient) AND variation (e.g. standard deviation) or associated estimates of uncertainty (e.g. confidence intervals)
☐	☒	For null hypothesis testing, the test statistic (e.g. F, t, r) with confidence intervals, effect sizes, degrees of freedom and P value noted<br>*Give P values as exact values whenever suitable.*
☒	☐	For Bayesian analysis, information on the choice of priors and Markov chain Monte Carlo settings
☒	☐	For hierarchical and complex designs, identification of the appropriate level for tests and full reporting of outcomes
☐	☒	Estimates of effect sizes (e.g. Cohen's d, Pearson's r), indicating how they were calculated

Our web collection on statistics for biologists contains articles on many of the points above.

Software and code

Policy information about availability of computer code

Data collection	*Provide a description of all commercial, open source and custom code used to collect the data in this study, specifying the version used OR state that no software was used.*
Data analysis	MATLAB R2020<br>R version 4.1.0

For manuscripts utilizing custom algorithms or software that are central to the research but not yet described in published literature, software must be made available to editors and reviewers. We strongly encourage code deposition in a community repository (e.g. GitHub). See the Nature Portfolio guidelines for submitting code & software for further information.

Data

Policy information about availability of data

All manuscripts must include a data availability statement. This statement should provide the following information, where applicable:

- Accession codes, unique identifiers, or web links for publicly available datasets
- A description of any restrictions on data availability
- For clinical datasets or third party data, please ensure that the statement adheres to our policy

Data is not publicly available due to privacy issues regarding clinical data. Data can be made available upon request to the corresponding authors on the condition that a formal data sharing agreement is made.

Field-specific reporting

Please select the one below that is the best fit for your research. If you are not sure, read the appropriate sections before making your selection.

☒ Life sciences ☐ Behavioural & social sciences ☐ Ecological, evolutionary & environmental sciences

For a reference copy of the document with all sections, see nature.com/documents/nr-reporting-summary-flat.pdf

Life sciences study design

All studies must disclose on these points even when the disclosure is negative.

Sample size	The data consist of 23 first-episode stroke patients (34-74 years old; mean age 57 years; 8 female) and 24 healthy sex-matched controls (33-65 years old; mean age 52 years; 10 female). Sample size was not chosen explicitly; instead, all available data was used for the analysis.
Data exclusions	Subjects 12 and 20 were excluded from the linear model assessing the impact of dominant-CST damage on state parameters & motor recovery because they did not have imaging or motor scores for the chronic time period.
Replication	Main analyses of the paper were repeated with k=5 clusters (as opposed to k=4 in the main paper). Group differences in fractional occupancy and dwell times in FPN+ were replicated across k=5. Likewise, the group differences in transition probability between MOTOR- and FPN+ were replicated in k=5. The marginal effect for the dwell time dominant-CST analysis was replicated in k=5 but not the interaction effect, nor either effect for fractional occupancy.
Randomization	N/A
Blinding	N/A

Reporting for specific materials, systems and methods

We require information from authors about some types of materials, experimental systems and methods used in many studies. Here, indicate whether each material, system or method listed is relevant to your study. If you are not sure if a list item applies to your research, read the appropriate section before selecting a response.

Materials & experimental systems

n/a	Involved in the study	
☒	☐	Antibodies
☒	☐	Eukaryotic cell lines
☒	☐	Palaeontology and archaeology
☒	☐	Animals and other organisms
☐	☒	Human research participants
☒	☐	Clinical data
☒	☐	Dual use research of concern

Methods

n/a	Involved in the study	
☒	☐	ChIP-seq
☒	☐	Flow cytometry
☐	☒	MRI-based neuroimaging

Human research participants

Policy information about studies involving human research participants

Population characteristics	The data consist of 23 first-episode stroke patients (34-74 years old; mean age 57 years; 8 female) and 24 healthy sex-matched controls (33-65 years old; mean age 52 years; 10 female).
Recruitment	The criteria for enrollment were as follows: fully obtained admission history (within 7 d after onset of symptoms), single infarction confined to the pons identified on MRI, and no other concomitant brain lesion or previous infarcts. The exclusion criteria were as follows: contraindications for MRI, unclear onset of symptoms, lesions outside the pons or extensive infarcts involving the midbrain or the medulla, recurrence infarction or secondary hemorrhage during follow-up, deafness and/or blindness, aphasia, or a visual field deficit.
Ethics oversight	*Identify the organization(s) that approved the study protocol.*

Note that full information on the approval of the study protocol must also be provided in the manuscript.

Magnetic resonance imaging

Experimental design

Design type	resting-state
Design specifications	5 longitudinal imaging and motor assessments; 7 days, 14 days, 30 days, 90 days, 180 days post-stroke.
Behavioral performance measures	Fugl-Meyer assessment (assessed twice per subject at each session and averaged); 33 tests with max of 2 points per test (2 = full motor performance). Maximum score is 66, all scores were normalized to the range [0, 100].

Acquisition

Imaging type(s)	functional, strucutral
Field strength	3T
Sequence & imaging parameters	Anatomical images were acquired using a sagittal MP-RAGE three-dimensional T1-weighed sequence (TR, 1600ms; TE 2.15ms; flip angle, 9°, 1.0 mm isotropic voxels, FOV 256 x 256). Each MRI session involved between two and four runs of task-free fMRI at 6 minutes each. Subjects were instructed to stay awake with their eyes open; no other task instruction was provided. Images were acquired using the gradient-echo echo-planar pulse sequence (TR, 3000ms; TE, 30ms; flip angle, 90°, 3 mm isotropic voxels).
Area of acquisition	whole brain

Diffusion MRI ☐ Used ☒ Not used

Preprocessing

Preprocessing software	ANTs, CONN
Normalization	Preprocessing of the longitudinal anatomical MRIs included affine registration of each subject's T1 scans to the baseline T1 scan, collapsing co-registered files to an average T1 and creation of a skull-stripped brain mask followed by manual editing and binarization of the hand-edited mask. The brain mask was then transformed back to each of the follow-up T1s in native space using the inverse registration acquired from the first step. This was followed by bias field correction of all the T1 scans, transformation of native-space bias field-corrected data back to baseline space, and the creation of an average bias field-corrected scan for each subject. Stroke lesion masks were hand-drawn on these transformed T1 scans by ADB and JEB. Structural normalization was performed with the ANTs toolbox.
Normalization template	MNI152
Noise and artifact removal	Preprocessing of the longitudinal functional MRIs was performed using the CONN toolbox, including functional realignment of volumes to the baseline volume, slice timing correction for alternating acquisition, segmentation and normalization, and smoothing with a 4 mm FWHM kernel. This was followed by a denoising protocol (CompCor) which regressed out the cerebrospinal fluid and white matter signal, as well as 24 realignment parameters (added first-order derivatives and quadratic effects). Temporal band-pass filtering (0.008 - 0.09 Hz), despiking and global signal removal regression were also performed.
Volume censoring	The first four frames of each BOLD run were removed. Frame censoring was applied to scans with a framewise displacement threshold of 0.5 mm along with its preceding scan.

Statistical modeling & inference

Model type and settings	multivariate; 3 fixed effects (change in dynamic parameter, CST-Damage, interaction of: (change in dynamic parameter *CST-Damage)
Effect(s) tested	*Define precise effect in terms of the task or stimulus conditions instead of psychological concepts and indicate whether ANOVA or factorial designs were used.*

Specify type of analysis: ☒ Whole brain ☐ ROI-based ☐ Both

Statistic type for inference (See Eklund et al. 2016)	N/A
Correction	FDR; alpha value 0.05 using fdr_bh MATLAB toolbox

Models & analysis

n/a	Involved in the study
☒	☐ Functional and/or effective connectivity
☒	☐ Graph analysis
☒	☐ Multivariate modeling or predictive analysis
